# Altered Carbon Partitioning Enhances CO_2_ to Terpene Conversion in Cyanobacteria

**DOI:** 10.34133/2022/9897425

**Published:** 2022-02-07

**Authors:** Man Li, Bin Long, Susie Y. Dai, James W. Golden, Xin Wang, Joshua S. Yuan

**Affiliations:** ^1^Synthetic and Systems Biology Innovation Hub, Texas A&M University, College Station, Texas 77843, USA; ^2^Department of Plant Pathology and Microbiology, Texas A&M University, College Station, Texas 77843, USA; ^3^Guangdong Provincial Key Laboratory of Microbial Culture Collection and Application, State Key Laboratory of Applied Microbiology Southern China, Institute of Microbiology, Guangdong Academy of ScienceChina; ^4^Section of Molecular Biology, University of California San Diego, La Jolla, CA 92093, USA; ^5^Department of Microbiology, Miami University, Oxford, Ohio 45056, USA

## Abstract

Photosynthetic terpene production represents one of the most carbon and energy-efficient routes for converting CO_2_ into hydrocarbon. In photosynthetic organisms, metabolic engineering has led to limited success in enhancing terpene productivity, partially due to the low carbon partitioning. In this study, we employed systems biology analysis to reveal the strong competition for carbon substrates between primary metabolism (e.g., sucrose, glycogen, and protein synthesis) and terpene biosynthesis in *Synechococcus elongatus* PCC 7942. We then engineered key “source” and “sink” enzymes. The “source” limitation was overcome by knocking out either sucrose or glycogen biosynthesis to significantly enhance limonene production *via* altered carbon partitioning. Moreover, a fusion enzyme complex with geranyl diphosphate synthase (GPPS) and limonene synthase (LS) was designed to further improve pathway kinetics and substrate channeling. The synergy between “source” and “sink” achieved a limonene titer of 21.0 mg/L. Overall, the study demonstrates that balancing carbon flux between primary and secondary metabolism can be an effective approach to enhance terpene bioproduction in cyanobacteria. The design of “source” and “sink” synergy has significant potential in improving natural product yield in photosynthetic species.

## 1. Introduction

Terpenoids are a large class of natural products with diverse biological functions, including photon harvesting (e.g., chlorophylls), membrane stability (e.g., sterols), and multitrophic signaling [1, 2]. Many terpenoids are also valuable chemicals with broad applications in the pharmaceutical, nutraceutical, cosmetic, and biofuel industries [3]. The past decade has witnessed a rapid increase in atmospheric CO_2_ levels due to fossil fuel combustion and deforestation. Photosynthetic terpene production represents a promising technology to mitigate global climate change by directly converting CO_2_ into hydrocarbon for “drop-in” biofuels, which could both reduce fossil fuel utilization and enable sustainable carbon capture and utilization [4, 5]. Moreover, photosynthetic organisms produce some of the most valuable terpenoid-derived medicines and vaccine adjuvants including taxol, artemisinin, vinblastine, and squalene [3]. The design of efficient CO_2_ conversion to terpenes in photosynthetic organisms thus has a broad industrial implication.

Despite significant efforts invested in improving photosynthetic terpene production, productivity is still limited. Cyanobacteria have recently emerged as a major model system for terpene production owing to their rapid growth and readily available genetic toolbox compared to eukaryotic algae and plants [6]. In cyanobacteria, terpenes are synthesized from two C_5_ precursors, dimethylallyl pyrophosphate (DMAPP) and isopentenyl pyrophosphate (IPP) [7, 8]. They are derived from the methylerythritol phosphate (MEP) pathway by sequentially condensing pyruvate and the photosynthate glyceraldehyde-3-phosphate (G3P) in seven enzymatic steps [9] (Figure S1). A recent study successfully validated computational modeling to enhance terpene productivity by overexpressing key downstream enzymes [8]. Terpene yields have also been improved by channeling pentose phosphate intermediates into the MEP pathway to create an alternative “source” [10] and design of a storage organelle to establish an artificial “sink” [11]. Despite this progress, terpene productivities need further improvement, which will depend on understanding fundamental mechanisms regulating terpene productivity.

The challenge for enhancing terpene productivity in a photosynthetic system remains to be the low carbon partitioning into the MEP pathway [12]. The estimated photosynthetic carbon partitioning for terpene synthesis is less than 1% in cyanobacteria [12]. Previous research has established that enhancing carbon “sink” capacity could synergize with upstream photosynthesis and increase product yield [13]. We also observed increased expression of carbon fixation enzymes in an engineered strain with high limonene productivity [8]. It is thus critical to understand additional metabolic and biochemical bottlenecks in the overall CO_2_-to-terpene conversion process to achieve improved carbon partitioning.

In this study, we employed systems biology approaches to uncover the limitations impeding CO_2_-to-terpene conversion in *Synechococcus elongatus* PCC 7942 (*S. elongatus*). Specifically, we revealed the carbon partitioning competition between carbohydrate and terpene biosynthesis. The systems biology study guided design of an efficient CO_2_-to-terpene conversion. Enhanced limonene production was achieved by tuning down the sucrose and glycogen biosynthesis pathways. The effectiveness of altered carbon partitioning was further improved by engineering downstream fusion enzymes to enhance “sink” capacity.

## 2. Materials and Methods

### 2.1. Experimental Design

**Figure 1:**
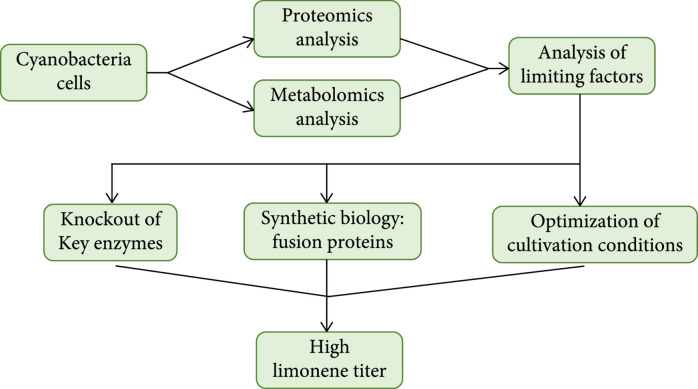
Overview of experimental scheme.

In order to improve limonene production in cyanobacteria (Figure 1), we first performed proteomics and metabolomics analyses to identify limiting factors in limonene production. We verified the systems biology outcome and knocked out several enzymes in pathways that compete with limonene biosynthesis for substrates. We also improved the downstream pathway kinetics by substrate channeling using fusion enzymes and optimized cultivation conditions for further limonene production enhancement.

### 2.2. Stain Growth Conditions

The limonene-producing *S. elongatus* strain L1118 [8] and newly engineered strains were grown in BG11 medium (Sigma, Chicago, IL) supplied with 10 mM N-[Tris(hydroxymethyl)methyl]-2-aminoethanesulfonic acid (TES, pH=8.2) at 30°C. The strains were first cultured in 250 mL flasks with illumination of 75 *μ*mol photons m^-2^ s^-1^ with the addition of appropriate antibiotics and then transferred into a 1-L Roux bottle with 5% CO_2_ bubbling at a speed of 50 mL/min and 100 *μ*mol photons m^-2^ s^-1^. For the optimized cultivation experiments, 50 mL of cyanobacteria was cultivated in a cylinder photobioreactor with 3 cm diameter. Incremental light intensities were set to 100 *μ*mol photons m^-2^ s^-1^ on day 0, 200 *μ*mol photons m^-2^ s^-1^ on day 1, 200 *μ*mol photons m^-2^ s^-1^ on two sides of the photobioreactors on day 2, and 300 *μ*mol photons m^-2^ s^-1^ on two sides of the photobioreactors thereafter. The temperature was increased to 37°C, and bubbling speed was increased to 1 L/min.

### 2.3. Plasmid and Strain Constructions

Strains and plasmids used in this study are listed in Table S1. The plasmids were constructed through Gibson Assembly (NEB, Ipswich, MA). The related genes/fragments were amplified from genomic DNA using Phusion High-Fidelity DNA Polymerase (NEB, Ipswich, MA). The L1118 and Lgpps-ls were constructed by integrating *ls* (from *Mentha spicata*) and fusion genes of *gpps-ls* into neutral site 1 of *S. elongatus*, respectively. Strains overexpressing *ispG*, *gap2*, and *pgk* were constructed by inserting the expression cassettes (driven by pLacO promoter) into neutral site 2. For *sps* and *glgC* knockout, coding regions of *sps* and *glgC* were replaced with gentamycin and kanamycin resistant genes, respectively.

### 2.4. Limonene Collection and Measurement by Gas Chromatography-Mass Spectrometry

*S. elongatus* strain L1118 and other engineered cyanobacterial cells were grown in 1-L Roux bottles coupled with a HayeSep polymer trap. The vaporized limonene was collected each day by eluting with 1 mL hexane containing 10 *μ*g/mL cedrene as the internal standard. Samples were analyzed by gas chromatography-mass spectrometry (GC-MS). GC-MS was performed on a GCMS-QP2010SE instrument (Shimadzu Scientific Instruments, Inc.). One-microliter eluted sample was injected into a Shimadzu SH-Rxi-5Sil column (30 m×250 μm×0.25 μm) using helium as carrier gas at the flow rate of 1.0 mL/min. The GC temperature profile was held at 50°C for 3 min and then increased to 140°C at 20°C/min. Mass spectral peak quantification was performed using GCMSolution software Ver. 2.6 against a standard curve.

### 2.5. Total Protein Extraction

50 mL cultures from each strain were harvested by centrifugation at 8000 rpm, 4°C for 10 min. The pellets were then washed with 10 mL of 0.9% NaCl. Cells were suspended in 2 mL 50 mM Tris-HCl, 10 mM CaCl_2_, and 0.1% Nonidet P-40 (pH=7.6) supplemented with 20 *μ*L protease inhibitor cocktail (Sigma, USA). Cells were lysed by tip-probe sonication through 20 cycles of sonication with 15 s on and 60 s off on ice. The lysates were centrifuged at 12000 rpm for 10 min at 4°C. Supernatants were collected and stored at -80°C for future proteomics. The protein concentration was measured through Pierce Bradford Protein Assay (Thermo Fisher) with standards following manufacturer’s instructions. Samples from each strain were prepared from three biological replicates.

### 2.6. Proteomics and Data Analysis

One hundred micrograms of total proteins from each sample were denatured with 8 M urea and 5 mM DTT and incubated at 37°C for 1 hour. The denatured proteins were then treated with iodoacetamide to a final concentration of 15 mM, followed by an incubation period of 15 min at room temperature in the dark. The samples were diluted 4 times to a final concentration of 2 M urea. One *μ*g trypsin (Promega, Madison, WI) was added into each sample and incubated at 37°C overnight. The digested peptide was desalted through a sep-pak C18 column (Waters, Milford, MA). A SpeedVac (Brinkmann Instruments, Westbury, NY) was used to lyophilize samples. The digested proteins were dissolved in 0.1% formic acid and centrifuged for 10 min at 12000 rpm at 4°C. The supernatant was harvested and stored at -80°C for future use. The desalted samples were loaded into an in-house packed 150 *μ*m capillary column. The mass spectra were collected with a LTQ mass spectrometer (Thermo Scientific) and searched against a composite database containing proteins of *S. elongatus*, common contaminants, and reversed sequences using the ProLuCID algorithm [14]. The output files were filtered through PatternLab 4.0 [15] for the following data analysis.

## 3. Results

### 3.1. Proteomic Analysis Reveals the Limitation of Substrate for Terpene Production

**Figure 2:**
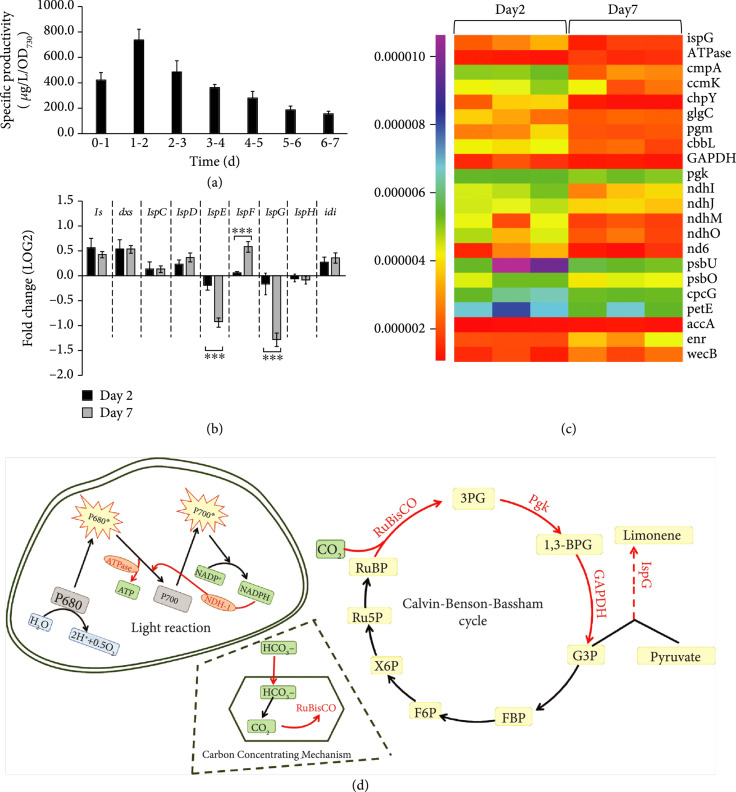
Proteomic analysis to reveal mechanisms for decreased limonene specific productivity in late growth stage. (a) Limonene specific productivity decreased after 2 days of cultivation. (b) Real-time PCR showed transcriptional changes of genes in the MEP pathway. Expressions of *dxs*, *IspE*, and *IspG* showed significant decreases on day 7. (c) Proteomics analyses revealed decreased expression of proteins involved in photosynthesis and terpene biosynthesis on day 7 as compared to day 2. Proteomics analysis was done with three biological replicates. Normalized spectral abundance factor (NSAF) [41] was used for the visualization and proteins shown in the figure have a P value of <0.05. Detailed information about NSAFs, fold changes, and P values of identified proteins is listed in dataset S1. (d) Scheme of pathway changes in L1118 on day 7. Red color refers to the related decrease in enzyme expression level. ndhI-O: NAD(P)H-quinone oxidoreductase subunit I-O; RuBisCO: ribulose-1,5-bisphosphate carboxylase/oxygenase; Pgk: phosphoglycerate kinase; GAPDH: glyceraldehyde 3-phosphate dehydrogenase; ispG: 4-hydroxy-3-methylbut-2-enyl diphosphate synthase. ${}^{**}P$ value < 0.05, ${}^{***}P$ value < 0.01.

In our previous study, limonene synthase (LS) was identified as a key flux-controlling step for limonene production in *S. elongatus* [8]. By creating a strong limonene sink through high *ls* gene expression (from *Mentha spicata*), the strain L1118 serves as an effective platform to investigate additional metabolic and biochemical limits in terpene biosynthesis. When L1118 cells were grown under continuous light for 7 days, we found significant variation in limonene productivity over time. The highest limonene specific productivity was achieved on the second day, followed by a continuous productivity decline (Figure 2(a)). The reductions could result from physiological changes due to reduced light availabilities and metabolic limitations at later growth stages [16].

In order to understand the metabolic limits for terpene production at different growth times, we first identified potential targets for additional engineering using quantitative reverse transcription PCR (RT-qPCR) analysis to evaluate the transcript levels of MEP pathway genes. The expression of *ispE* and *ispG* decreased significantly on day 7 compared to day 2. Interestingly, *ispF* expression was significantly higher on day 7, when limonene productivity was low. The expression of other MEP genes and *ls* showed similar levels between day 2 and day 7 (Figure 2(b)).

We carried out proteomics on the same two samples of L1118 cells. Among an average of 920 detected proteins (*S. elongatus* proteome contains 2657 proteins), IspG was the only MEP enzyme that showed differential expression between day 2 and day 7 with statistical significance (P<0.05). We thus overexpressed the *ispG* gene under the control of the LacO-1 promoter in L1118. The *ispG* gene from either *Synechocystis* sp. PCC 6803 or *Botryococcus braunii* was overexpressed in L1118 to generate strains L1218 and L1219, respectively. This overexpression led to significantly lower limonene productivity in the first two days (Figure S2A and B), indicating either sufficient IspG activity in L1118 or a limited role of IspG in enhancing MEP flux alone. Another possibility is that the increased IspG resulted in accumulations of IPP and DMAPP, which led to a feedback inhibition of 1-deoxy-D-xylulose-5-phosphate synthase (DXS), the first enzyme in the MEP pathway [17]. A previous study reported that isoprene production in *S. elongatus* increased as a result of cooverexpression of IspG and two other MEP enzymes (DXS and IDI) [18]. However, these two enzymes did not show differential expression in the case of limonene production. Together, these results show the ineffectiveness of engineering MEP genes to further enhance limonene production.

Further analysis of the proteomic results identified the G3P and pyruvate supply as a potential limiting factor for limonene production. The abundance of glyceraldehyde-3-phosphate dehydrogenase (GAPDH), the enzyme-converting 1,3-bisphosphoglycerate (1,3-BPG) to G3P, and phosphoglycerate kinase (PGK) was found to be significantly lower in L1118 cells at day 7 compared to day 2 (Figure 2(c)). The decreased expression of GAPDH and PGK indicates a lower photosynthetic carbon output (G3P) in the later growth stage. Moreover, enzymes involved in CO_2_ assimilation showed decreased expression on day 7 (Figure 2(c)). The levels of CO_2_ hydration protein ChpY and bicarbonate-binding protein CmpA showed 2.3-fold and 2.7-fold reductions, respectively (Figure 2(c)). As these two enzymes play an essential role in the CO_2_ concentrating mechanism (Figure 2(d)) [19], the reductions imply reduced carbon assimilation, which could further impact the G3P and pyruvate supply.

Coincidently, several proteins involved in the light reactions also showed decreased expression on day 7. Compared to protein levels on day 2, PS II extrinsic proteins (PsbU and PsbO) showed an average 1.4-fold reduction, and NAD(P)H-quinone oxidoreductase subunits I, J, M, O of the NDH-I complex proteins showed an average 2-fold decrease on day 7 (Figure 2(c) and Table S2). PS II extrinsic proteins protect the catalytic center for water oxidation during photosynthesis [20]. The NDH-I complex also plays an essential role in the cyclic electron flow of photosynthesis [21]. Together, the decreased expression of proteins involved in light reactions, the CO_2_ concentrating mechanism, and the Calvin-Benson cycle suggests a decreased photosynthetic efficiency on day 7 as compared to day 2, thus limiting the overall carbon output from photosynthesis (Figure 2(d)).

Interestingly, neither increasing substrate supply by feeding pyruvate and glycerol (Figure S2C and D) nor enhancing G3P production by overexpressing GAPDH and PGK (Figure S3) improved limonene productivity, suggesting additional bottlenecks in limonene biosynthesis. To further understand these limiting factors, we performed comparative metabolomics to investigate metabolism dynamics between different growth stages.

### 3.2. Metabolomics Reveals the Active Competition for Substrates

**Figure 3:**
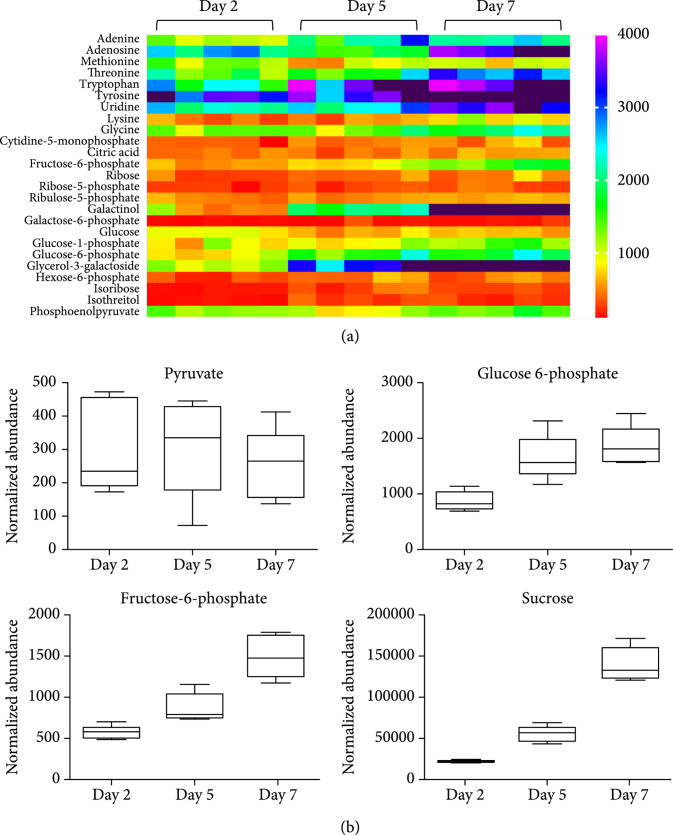
Metabolomic analysis reveals MEP flux limitations. (a) Overview of metabolomics at day 2, day 5, and day 7. The accumulation of amino acids and carbohydrate intermediates indicate an active substrate competition between primary metabolism and terpene biosynthesis. Detailed normalized abundances of identified metabolites are listed in dataset S1. (b) Detailed metabolite changes at day 2, day 5, and day 7. No significant changes were observed in pyruvate abundance at three different time points, while the abundances of glucose 6-phosphate, fructose 6-phosphate, and sucrose increased over time.

L1118 cells from day 2, day 5, and day 7 were collected for metabolomic analysis (see supplementary information for details). The results highlighted the changes in carbon partitioning toward primary metabolism during the stationary phase. First, the metabolomic analysis showed a significant increase of amino acids such as tyrosine and tryptophan on day 5 and day 7 (Figure 3). These aromatic amino acids are synthesized from erythrose 4-phosphate (E4P) and phosphoenolpyruvate (PEP) [22]. E4P serves as both an intermediate and an output of the Calvin-Benson cycle, whereas PEP can be derived from pyruvate. The accumulation of these amino acids indicates potential competition between amino acid biosynthesis and terpene biosynthesis for metabolite precursors at later growth stages.

Second, the metabolomic analysis also revealed the changes in carbon partitioning competition between carbohydrate metabolism and terpene metabolism. Sugars and sugar phosphates were another group of metabolites accumulated to a higher amount on day 7 (Figure 3(a)). The level of glucose-6-phosphate (G6P), fructose-6-phosphate (F6P), and sucrose increased significantly over the growth (Figure 3(b)). The increased levels of these metabolites over the cyanobacterial growth stages indicate photosynthate partitioning toward storage carbon when cells approach high densities. Interestingly, pyruvate levels were similar during these growth stages (Figure 3(b)), suggesting that it might not be a limiting substrate for the MEP pathway. The observation partially explains the feeding experiment results, where pyruvate supplementation failed to enhance limonene production (Figure S2C and D). Overall, the comparative metabolomics suggest an active substrate competition between terpene biosynthesis and primary metabolism, especially at later growth stages, which potentially result in decreased specific limonene productivity.

### 3.3. Carbon Partitioning from Sucrose Biosynthesis to Limonene Production

**Figure 4:**
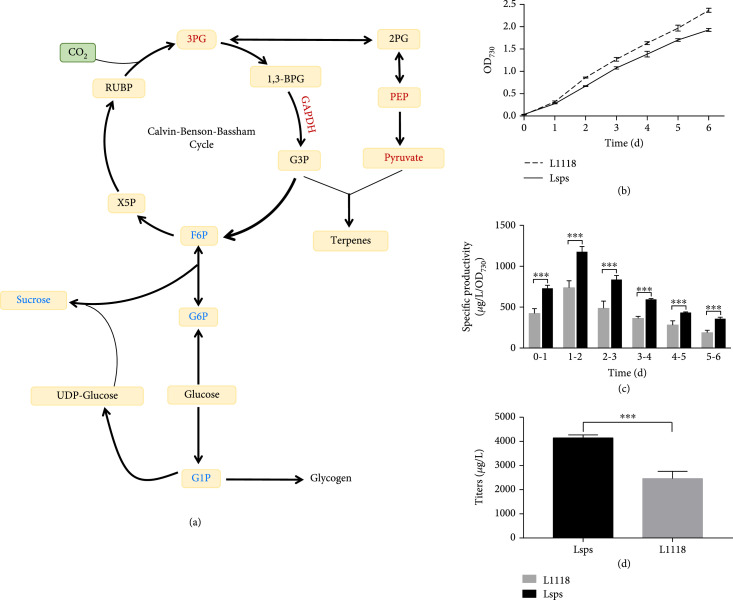
Blocking sucrose biosynthesis enhanced limonene production. (a) The comparative proteomics and metabolomics revealed increased levels of intermediates (red) for sucrose and glycogen biosynthesis, while the substrate supplies for terpene biosynthesis were decreased (blue). (b) Growth curve of Lsps and L1118 strains. Lsps showed slower growth compared to L1118. (c) Limonene specific productivity of Lsps and L1118. Lsps showed a significant increase in limonene specific productivities compared to L1118. (d) Lsps achieved significantly higher limonene titer compared to L1118. ${}^{***}P$ value < 0.01.

Proteomic and metabolomic analyses indicated a tight regulation in carbon flux distribution between the MEP pathway and other central metabolic pathways (Figure 4(a)). The limited capacity of the MEP pathway as a photosynthetic carbon sink calls for altering carbon partitioning from primary metabolism to terpene biosynthesis. The metabolomic analysis highlighted the accumulation of sucrose in the later growth stages. Sucrose is synthesized from photosynthates through two enzymatic steps. Specifically, UDP-glucose and fructose-6-phosphate (F6P) are first converted to sucrose-6-phosphate by sucrose-phosphate synthase (Sps), followed by sucrose phosphatase converting sucrose-6-phosphate into sucrose [23]. To achieve altered carbon partitioning from sucrose to terpene biosynthesis, we generated a sucrose synthesis mutant ∆*sps* (strain Lsps).

Compared to strain L1118, the sucrose mutant Lsps showed slightly lower biomass accumulation (Figure 4(b)). However, limonene specific productivity and titer were significantly higher in the Lsps strain compared to L1118 (Figures 4(c) and 4(d)). Specifically, the limonene-specific productivity in strain Lsps increased to 1100 *μ*g/L/OD/day compared to that of 885 *μ*g/L/OD/day in L1118 (Figure 4(c)), with the titer reaching 4.1 mg/L after six days of cultivation compared to 3.0 mg/L in L1118 (Figure 4(d)). Furthermore, no significant amount of sucrose was detected in Lsps (Figure S4), confirming that the deletion of the *sps* gene efficiently blocked sucrose biosynthesis and effectively redirected carbon flux to limonene biosynthesis. Altering carbon partitioning between sugar and terpene metabolism thus represents an effective approach to improve terpene productivity.

### 3.4. A Rewired Carbon Metabolism Supported Enhanced Limonene Production

A second comparative proteomics was carried out to investigate the protein profile changes in Lsps as compared to L1118. Several photosynthesis-related proteins were downregulated in the Lsps strain (Table S3), including photosystem II reaction center proteins (CP47, CP42, and D2), ATPase, and NAD(P)H-quinone oxidoreductase subunits (Ndh K, M, and N). Such decreases suggest a reduced photosynthetic rate that potentially led to the decreased biomass accumulation in the Lsps strain (Figure 4(b)).

**Figure 5:**
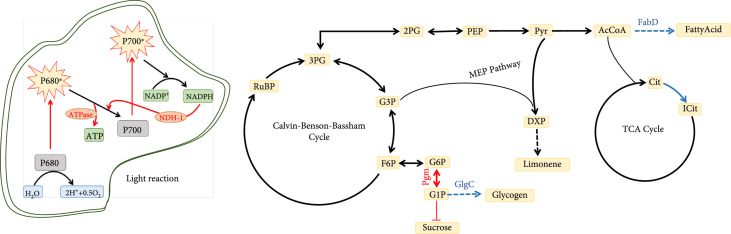
Comparative proteomics revealed the metabolism changes in Lsps compared to L1118. The expression levels of proteins involved in photosynthesis and sucrose biosynthesis were found to be lower (red) in Lsps compared to L1118. In contrast, other primary metabolism-related enzymes (blue) such as GlgC and FabD were higher in Lsps.

Sucrose is synthesized from glucose-1-phosphate (G1P). In Lsps, the expression of phosphoglucomutase, the enzyme catalyzing the conversion between G1P to G6P, decreased 1.4-fold compared to the L1118 strain (Figure 5 and Table S3). Moreover, the expression of glucose-1-phosphate adenylyltransferase (AGPase) for glycogen biosynthesis pathways increased 1.4-fold in Lsps, indicating a potential increase of glycogen accumulation. Enzymes involved in the fatty acid biosynthesis pathway were changed as well. Levels of 3-oxoacyl-(Acyl-carrier protein) reductase increased, whereas 3-oxoacyl- (Acyl-carrier protein) synthase decreased in the Lsps strain relative to the L1118 strain. Overall, by blocking the sucrose biosynthesis pathway in Lsps, photosynthetic outputs were likely redirected from sucrose biosynthesis to other metabolic processes such as the MEP pathway for limonene synthesis, fatty acid biosynthesis, and glycogen storage.

### 3.5. Rechanneling Carbon from Glycogen Biosynthesis to Limonene Production

**Figure 6:**
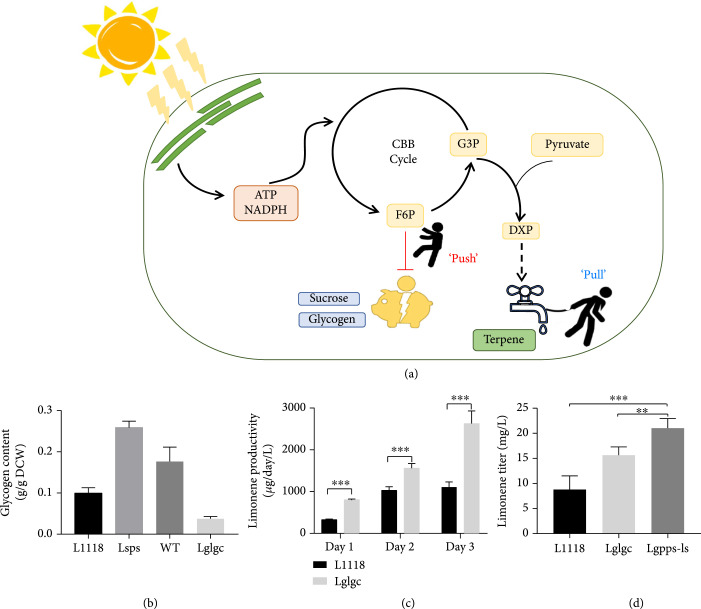
“Push” and “pull” strategy significantly enhanced limonene production. (a) Scheme showing the “push” and “pull” strategy. “Push” strategy was achieved by knocking out *glgc* for glycogen biosynthesis while the “pull” strategy was realized with the GPPS-LS fusion design. (b) Measurement of glycogen content in WT, L1118, Lsps, and Lglgc strains. Lsps showed highest glycogen content, indicating the carbon redistribution from sucrose to glycogen biosynthesis. The low glycogen content in Lglgc verified its deficiency in glycogen synthesis. (c) Limonene productivity in L1118 and Lglgc. Lglgc showed significant higher limonene productivity than L1118. (d) 5-day limonene titer comparison between L1118, Lglgc, and Lgpps-ls. The Lgpps-ls accumulated 21.0 mg/L limonene in 5 days. ${}^{**}P$ value < 0.05; ${}^{***}P$ value < 0.01.

As the proteomics results revealed potential carbon channeling from sucrose to glycogen biosynthesis in Lsps, we compared the glycogen content in *S. elongatus* wild type, L1118, and Lsps. Consistent with the proteomics results, a significant increase of glycogen was observed in Lsps cells compared to L1118 and wild-type cells (Figure 6(b)). Interestingly, the glycogen content of L1118 was significantly lower than that of the wild type (Figure 6(b)), presumably due to competition from limonene biosynthesis. The results suggest glycogen as an additional strong carbon sink after blocking sucrose biosynthesis. In *S. elongatus*, three enzymes, AGPase, glycogen synthase (GS), and 1,4-alpha-glucan branching enzyme (GBE1), are involved in glycogen biosynthesis from G1P. Deleting the AGPase gene *glgC* can successfully block glycogen biosynthesis in *S. elongatus* and *Synechocystis* sp. PCC 6803 [24, 25]. Our proteomics data showed high expression levels of AGPase in Lsps cells (Table S3). To further enhance limonene production, we blocked glycogen biosynthesis by knocking out *glgC*. The *glgC* gene was replaced with a kanamycin-resistance gene in both L1118 and Lsps, creating Lglgc and Lglgc-sps (sucrose and glycogen double knockout strain), respectively.

The Lglgc-sps strain showed significant reductions in both growth and limonene productivity compared to Lglgc (Figure S5A and B), indicating that carbohydrate metabolism is critical in maintaining normal cyanobacterial growth. Indeed, ATP concentration in Lglgc-sps was found to be over 2-fold higher compared to L1118 and Lglgc (Figure S5C). The excess ATP might indicate low rates of carbon fixation and other ATP-consuming metabolic processes, which eventually impede cell growth. In contrast, Lglgc showed significant improvement in limonene productivity, achieving 2.46 and 1.52-fold increases compared to L1118 on day 1 and day 2, respectively (Figure 6(c)). The glycogen content in Lglgc was significantly lower than in other strains (Figure 6(b)). The results suggest that carbon flux was rechanneled from primary metabolism to limonene production by blocking glycogen biosynthesis. Interestingly, a previous study suggested that blocking glycogen biosynthesis does not lead to increased limonene production in *Synechococcus* sp. PCC 7002 [26], which might be related to differences in strain background and cultivation system.

### 3.6. Cultivation Optimization to Achieve Higher Limonene Yields

Although significant limonene productivity increases were achieved in both Lsps and Lglgc strains, the limonene production potentials in both strains are not fully realized due to light inhibition and nonoptimal cultivation conditions. The near-linear growth of cyanobacterial strains indicates light limitation during cultivation [27] (Figure S2A). To adapt to light demand changes throughout cultivation, we incremented light intensities and reduced the photobioreactor size for better light penetration. Additionally, the growth temperature was increased from 30°C to 37°C, the optimal growth temperature for *S. elongutas*, according to a recent publication [28]. By optimizing growth conditions, the 5-day limonene titers of L1118 and Lglgc increased to 8.8 mg/L and 15.6 mg/L, respectively (Figure 6(d)).

### 3.7. Biodesign of “Push” and “Pull” Strategy to Further Enhance Limonene Productivity

In addition to cultivation optimization, we also sought to further improve limonene production through a “push” and “pull” strategy (Figure 6(a)). In particular, the *glgC* knockout represents a “push” strategy to enhance carbon partitioning to MEP by blocking competing pathways. Because the proteomics results revealed that limonene synthase is the top third most abundant protein among all detected proteins (Figure S6), we ascertain that limonene synthase (LS) abundance is less likely to be the limiting factor in limonene production. However, the enzymatic kinetics of LS could be enhanced by increasing the local concentration of its substrate, geranyl diphosphate (GPP), as described by a previous study [18]. In cyanobacteria, GPP is converted from MEP outputs (DMAPP and IPP) by geranyl diphosphate synthase (GPPS). Therefore, we designed a “pull” strategy by fusing GPPS from *Abies grandis* to the N-terminal of LS, with a GGGS linker in between. The fusion-enzyme design could enhance substrate channeling and increase limonene yield. Because Lglgc showed higher productivity than Lsps, the plasmid with the fused GPPS-LS enzymes was transformed into the Lglgc strain, generating Lgpps-ls. The engineered strain synergized the “push” and “pull” strategy, where the “source” for terpene synthesis was increased *via* altered carbon partitioning, and the “sink” for terpene synthesis was enhanced *via* substrate channeling. Under the optimized growth condition, the “push” and “pull” design led to a 1.3-fold increase in limonene titer, reaching 21.0 mg/L in 5 days (Figure 6(d)), which is the highest among all published results in cyanobacteria (Table 1). Overall, our results not only support the effectiveness of the altered carbon partitioning strategy but also highlight the need to make MEP and downstream terpene biosynthesis more efficient to achieve increased carbon partitioning to terpene production.

**Table 1:** Limonene titers from different studies.

Species	Genotypes	Titers	Years	References
*Synechocystis* PCC 6803	P*trc*::*dxs-crtE-idi*, P*trc*::*ls*	1 mg/L	2014	[29]
*Synechococcus* PCC 7002	P*cpc*::*ls*	4.0 mg/L	2014	[26]
*Synechococcus* PCC 7942	P*psbA*::*ls*	2.5 mg/L	2016	[8]
*Synechocystis* PCC 6803	P*trc1O*::*ls*, P*rbcL*::*rpi-rpe-gpps*	6.7 mg/L	2017	[10]
*Cyanothece* PCC 7425	P*λpR*::*ls*	0.8 mg/L	2020	[34]
*Escherichia coli* BL21	*MVA*, P*trc*::*gpps-ls*	3.6 g/L	2020	[31]
*Synechococcus* UTEX 2973	P*trc1O*::*ls-gpps*, P*lacUV5*::*dxs-idi*	16.4 mg/L	2021	[39]
*Saccharomyces cerevisiae*	P*adh2::acs*, P*gal10::npps*, P*galj::ls*, P*gal10:: idi*	2.23 g/L	2021	[40]
*Synechococcus* PCC 7942	*Δglgc*, P*psbA*::*ls-gpps*	21.0 mg/L	2022	This study

## 4. Discussion

In this study, we employed a systems biology strategy to study carbon partitioning between primary and secondary metabolism in cyanobacteria, opening new avenues for engineering production of natural products at higher productivities. Several recent studies have engineered cyanobacteria to produce limonene, but productivities are still low compared to heterotrophic systems (Table 1) [8, 10, 26, 29–31]. Using proteomic and metabolomic analyses, this study reveals that primary metabolism, including biosynthesis of amino acids, fatty acids, and carbohydrates, was upregulated at the late growth stage (day 7) when limonene production was decreased. The shift of carbon partitioning between primary and secondary metabolisms likely results from photophysiology alterations due to growth phase changes [27]. Specifically, enhanced primary metabolism competes for substrates with secondary metabolism, leading to low limonene productivity. This understanding guided altered carbon partitioning towards terpene biosynthesis by knocking out key enzymes in primary metabolism, leading to improved limonene yields.

The experimental results verified our hypothesis and can be used to guide future engineering. Blocking sucrose and glycogen biosynthesis significantly increased limonene productivity, supporting the idea that competition for the substrates from primary metabolism is a “source” limiting factor for terpene production in cyanobacteria. However, limonene productivity increases were not proportional to the decreases of either sucrose in the Lsps strain or glycogen in the Lglgc strain. Instead, the subsequent proteomic analysis found that other primary metabolisms, including fatty acid biosynthesis and glycogen biosynthesis, were upregulated in Lsps relative to L1118. Correspondingly, the glycogen content in Lsps was found to be 1.5-fold higher than in L1118 (Figure 6(b)). These observations suggest that a larger portion of carbon flux was shunted to other primary metabolism pathways instead of terpene biosynthesis in the Lsps and Lglgc strains. The results also corroborate previous findings that blocking glycogen biosynthesis results in a high level of alpha-ketoglutaric acid production in response to increased energy charge [32]. Overall, the results highlight the idea that altered carbon partitioning can only be achieved through synergizing with a more effective “sink” capacity for terpene synthesis.

In fact, these results suggest the “sink” as potential limiting factor in limonene production in addition to the “source” limitation. Increasing G3P and pyruvate successfully enhanced isoprene production in *E. coli* [33]. However, neither *in vitro* supplement of G3P and pyruvate (Figure S2C and D) or *in vivo* overexpression of GAPDH and PGK (Figure S3) led to increased limonene production. Moreover, although altering carbon partitioning from primary metabolism was demonstrated as an effective strategy to enhance limonene production, only a small portion of carbon was rechanneled from either sucrose or glycogen to terpene. Therefore, “sink” improvement, together with altered carbon partitioning, was developed as a promising strategy to improve photosynthetic terpene production. We have shown that increasing pathway kinetics and substrate channeling by fusing GPPS with LS successfully improved limonene productivity in the LglgC strain (Figure 6(d)). By overcoming the limitations in “source” and “sink” through “push” and “pull” strategies, we achieved a record limonene titer among all of the engineered cyanobacterial strains [10, 26, 30, 34]. In addition to the last two steps of limonene biosynthesis, the omics analyses indicated IspG as another potential limiting factor in limonene production. However, overexpression of IspG failed to increase limonene productivity, suggesting the presence of other limitations. These limitations could include other enzymes in the MEP pathway or the electron donor for IspG; IspG is a [4Fe-4S] cluster-containing protein and requires a unique redox donor for its activity [35]. Moreover, the “sink” capacity could be further enhanced by introducing the mevalonate pathway (MVA) into cyanobacteria in parallel with the endogenous MEP pathway [7]. Nevertheless, the results demonstrated the importance of synergizing “source” and “sink” approaches to achieve more efficient carbon partitioning from primary to secondary metabolism.

While it is critical to synergize the “push” and “pull” strategies, it is also important to adopt strategies to mitigate potential detriments to cell physiology when engineering primary metabolism for altering carbon partitioning. Many primary metabolism pathways are essential in maintaining cell physiology and growth. In this study, both Lsps and Lglgc strains showed slightly lower biomass accumulation compared to L1118, and the double knockout of *Sps* and *glgC* together resulted in severe growth inhibition (Figure 4(b), S5A, and S5D). The slower growth of Lsps might result from the lower rate of net photosynthesis, as many photosynthesis-related proteins were found to be downregulated in this strain. The abolishment of glycogen metabolism may have interfered with energy buffering and Calvin-Benson cycle stabilization [32, 36], which could eventually lead to slower growth. Moreover, glycogen metabolism also contributes greatly to cell fitness in cyanobacterial diel growth [37]. Cell physiology and strain fitness thus need to be taken into consideration in future engineering efforts. One of the strategies to mitigate adverse impacts on cell physiology is to knock down the expression of *Sps* and *glgC* instead of knocking out these genes. Such a strategy can be further integrated with a stronger downstream terpene “sink” to achieve a higher terpene yield. Overall, the systems biology analysis and experimental validation in this study provided new insights into how to engineer higher production of natural products for broad applications.

## Data Availability

The mass spectrometry proteomics data have been deposited to the ProteomeXchange Consortium via the PRIDE [38] partner repository with the dataset identifier PXD030282.

## Supplementary Materials

Supplementary Materials Supplementary Figures S1-S6. Figure S1: pathway map for limonene synthesis in L1118. Figure S2: effects of MEP pathway engineering and substrate feeding on cell growth and limonene production. Figure S3: specific limonene productivity of engineered GAPDH and PGK strains. Figure S4: sucrose measurement by HPLC. Figure S5: phenotypes of glgC and sps mutants. Figure S6: relative abundance of limonene synthase (LS) detected by proteomics. Table S1: strains and plasmids used in this study. Table S2: enzyme expression level changes in log phase and stationary phase in L1118. Table S3: proteomics analysis of strain Lsps comparing to L1118. Supplementary Files S1-S5. File S1: plasmid map and sequence of pLM21. File S2: plasmid map and sequence of pWX1218. File S3: plasmid map and sequence of pWX1219. File S4: plasmid map and sequence of pWX1221. File S5: plasmid map and sequence of pLM2211.
