# Supplementary material for: Altered Carbon Partitioning Enhances CO2 to Terpene Conversion in Cyanobacteria
Source: Biodes Res. 2022 Feb 7;2022:9897425. doi: 10.34133/2022/9897425 (PMC10521692; doi:10.34133/2022/9897425)
Supplement: Supplementary Materials — Supplementary Figures S1-S6. Figure S1: pathway map for limonene synthesis in L1118. Figure S2: effects of MEP pathway engineering and substrate feeding on cell growth and limonene production. Figure S3: specific limonene productivity of engineered GAPDH and PGK strains. Figure S4: sucrose measurement by HPLC. Figure S5: phenotypes of glgC and sps mutants. Figure S6: relative abundance of limonene synthase (LS) detected by proteomics. Table S1: strains and plasmids used in this study. Table S2: enzyme expression level changes in log phase and stationary phase in L1118. Table S3: proteomics analysis of strain Lsps comparing to L1118. Supplementary Files S1-S5. File S1: plasmid map and sequence of pLM21. File S2: plasmid map and sequence of pWX1218. File S3: plasmid map and sequence of pWX1219. File S4: plasmid map and sequence of pWX1221. File S5: plasmid map and sequence of pLM2211. [file 9897425.f1.zip › Supplementary_v5.docx]

# **Supplementary Information for**

**Altered carbon partitioning enhances CO_2_ to terpene conversion in cyanobacteria**

Man. Li^1,2,3,^ ^†^, Bin. Long ^1,2, †^, Susie. Y. Dai^2^, James. W. Golden^5^, Xin. Wang^1,2,4*^, and Joshua. S. Yuan^1,2*^

^1^ Synthetic and Systems Biology Innovation Hub, Texas A&M University, College Station, Texas 77843, United States

^2^ Department of Plant Pathology and Microbiology, Texas A&M University, College Station, Texas 77843, United States

^3^ Current Address: Guangdong Provincial Key Laboratory of Microbial Culture Collection and Application, State Key Laboratory of Applied Microbiology Southern China, Institute of Microbiology, Guangdong Academy of Science

^4^ Department of Microbiology, Miami University, Oxford, Ohio 45056, United States

^5^ Section of Molecular Biology, University of California San Diego, La Jolla, CA 92093, United States

† Man Li and Bin Long contributed equally to this article. Author order was determined by the corresponding authors.

*Correspondence

Joshua S. Yuan: syuan@tamu.edu

Xin Wang: [xwang@miamioh.edu](mailto:xwang@miamioh.edu)

Supplementary Material and Methods

Quantitative reverse transcription PCR (RT-qPCR)

*S. elongatus* strain L1118 cells were grown in 500 mL BG11 (Sigma, USA) in a 1-L Roux bottle with 5% CO_2_ bubbling and 100 μmol photons m^-2^ s^-1^ for 7 days. 10 mL of the cyanobacteria cells were immediately chilled in an ice bath before being centrifuged at 5000 rpm for 10 min at 4 ºC, followed by total RNA extraction using TRIzol reagent. Two µg of RNA sample was first treated with DNase I (Sigma, #AMPD1) following the manufacturer’s instruction. The cDNA synthesis was performed in a 20 µL reaction using the OneStep RT-PCR kit (Qiagen, USA) following the manufacturer’s instruction.

All of the MEP pathway genes and the limonene synthesis gene were analyzed to compare their expression levels between the two growth stages. The sigma factor *RpoD* gene was used as the internal control. The synthesized cDNA was diluted 500 times and used as the template for qPCR using the 2 × SYBR green master mix (Bio-Rad, USA). For each gene, 2 technical replicates and 3 biological replicates were included, and the real-time PCR was carried out on a Bio-Rad Real-time PCR system (Bio-Rad, USA). For gene expression comparison, ΔCt was calculated as the cycle difference between the target gene and the *rpoD* control. The average of ΔΔCt, calculated by subtracting each ΔCt with the average ΔCt of one of the biological replicates for each gene, was used to compare the fold change of gene expression between the two growth stages. A ΔΔCt value close to 0 means minimal change between replicates.

ATP determination in *S. elongatus*

1.5 mL cultures of *S. elongatus* engineered strains were collected and harvested by centrifugation at 13,000 rpm for 5 mins. The pellets were washed with 0.9% NaCl twice. 100 µL prechilled 1% TCA was added to each sample. The samples were vortexed for 30s and then centrifuged at 4 °C for 10 mins. 100 µL of the supernatant was collected and neutralized with 100 µL of 1 M Tris-acetate pH=7.8. 800 µL of distilled water was then added to each tube. An ATP Determination Kit (Molecular Probes) was used for ATP measurement. 10 µL of the previously prepared sample was added to each well of a Corning 96-Well Black Plate (Sigma Aldrich) to reduce crossover noise. 10 µL ATP standards with concentrations of 50, 100, 250, 500, and 1000 nM/mL were measured together with the samples. The standard reaction solution containing luciferase and D-luciferin was prepared following the manufacturer’s instruction. 100 µl of reaction solution was added to each well and gently mixed with the samples. The luminescence was collected and ATP concentration was calculated on a SpectraMax i3x (Molecular Devices).

Comparative metabolomics and sugar analysis by HPLC

Comparative metabolomic analysis was done by the West Coast Metabolomics Center, Davis, CA, as described previously (1, 2). In general, five biological samples at each growth stage were analyzed and each metabolite’s abundance was normalized to the cell count, determined by using a cell imaging system (SpectraMax i3x, Molecular Device). For sucrose analysis, 50 mL cells from each strain were collected by centrifugation at 8000 rpm, 4 °C for 10 min. The pellets were then lyophilized using a 4.5 L Benchtop Freeze-Dry System (Labconco Corp., USA). Three mL of 80% ethanol was added to each sample for extraction of sucrose. The samples were heated at 65 °C for 3 h and then cooled at 4 °C overnight. Then the samples were centrifugated at 13000 rpm, 4 °C for 10 min. The supernatant was collected and dried in a SpeedVac (Thermo, USA). The dried samples were dissolved in 1 mL HPLC grade water and then analyzed with an HPLC Ultimate 3000 (Thermo, USA) equipped with an Aminex HPX-87P carbohydrate analysis column (Bio-Rad, USA) with a refractive index detector at 85 °C. HPLC grade water was used as the mobile phase for HPLC analysis at a flow rate of 0.6 mL/min. Glycogen was extracted from lyophilized cells using 30% KOH and measured with 0.2% anthrone reagent.

# **Supplementary Figure S1-S6**

**Figure S1.** Pathway map for Limonene synthesis in L1118.

**Figure S2.** Effects of MEP pathway engineering and substrate feeding on cell growth and limonene production.

**Figure S3.** Specific limonene productivity of engineered GAPDH and PGK strains.

**Figure S4.** Sucrose measurement by HPLC.

**Figure S5.** Phenotypes of *glgC* and *sps* mutants.

**Figure S6.** Relative abundance of limonene synthase (LS) detected by proteomics.


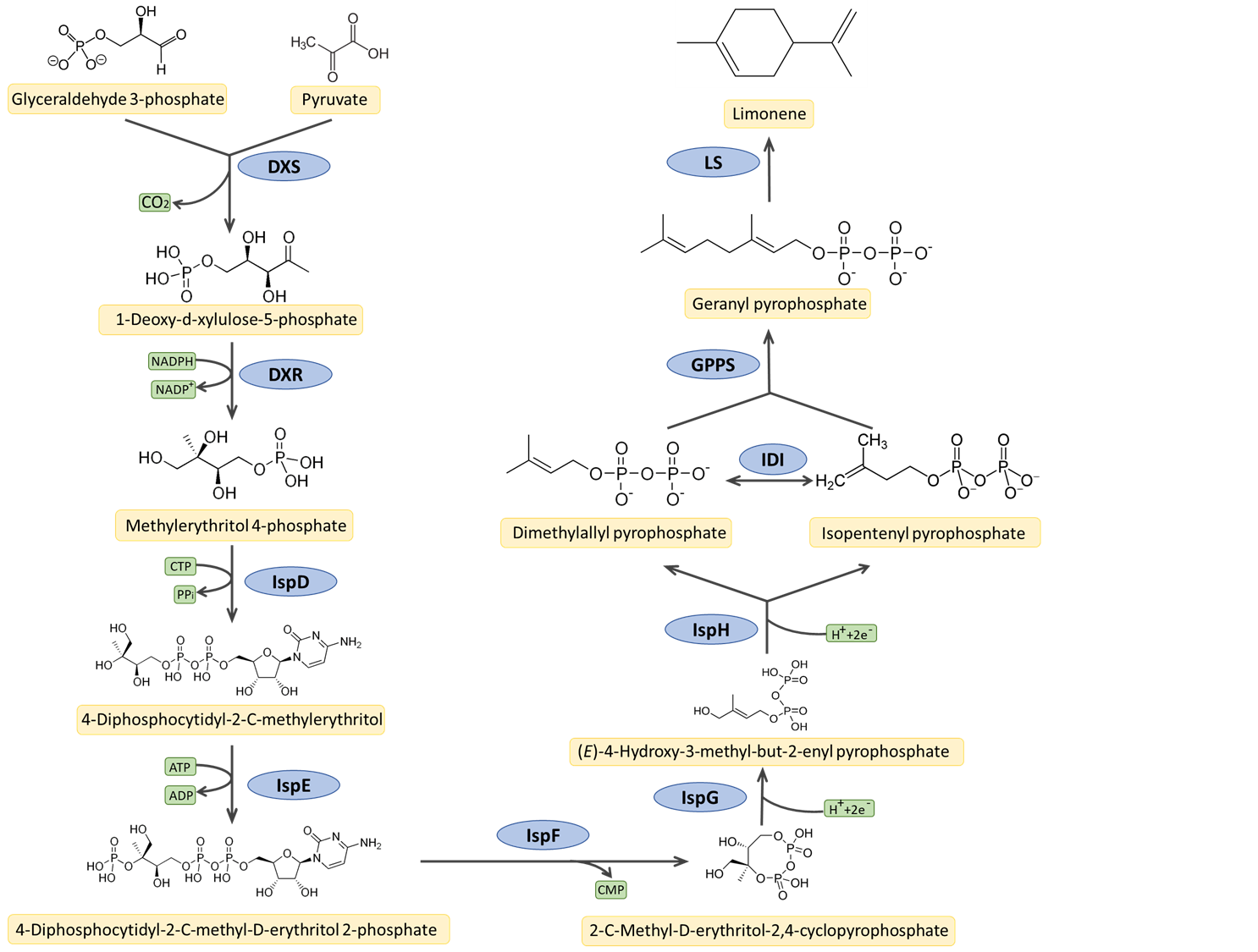


## **Figure S1.** Pathway map for Limonene synthesis in *Synechococcus elongatus* L1118. DXS: 1-deoxy-D-xylulose-5-phosphate synthase; DXR: 1-deoxy-D-xylulose 5-phosphate reductoisomerase; IspD: 2-C-methyl-D-erythritol 4-phosphate cytidylyltransferase; IspE: 4-diphosphocytidyl-2-C-methyl-D-erythritol kinase; IspF: 2-C-methyl-D-erythritol 2,4-cyclodiphosphate synthase; IspG: 4-hydroxy-3-methylbut-2-en-1-yl diphosphate synthase; IspH: 4-hydroxy-3-methylbut-2-enyl diphosphate reductase; IDI: Isopentenyl-diphosphate delta-isomerase; GPPS: Geranyl pyrophosphate synthase; LS: Limonene synthase.


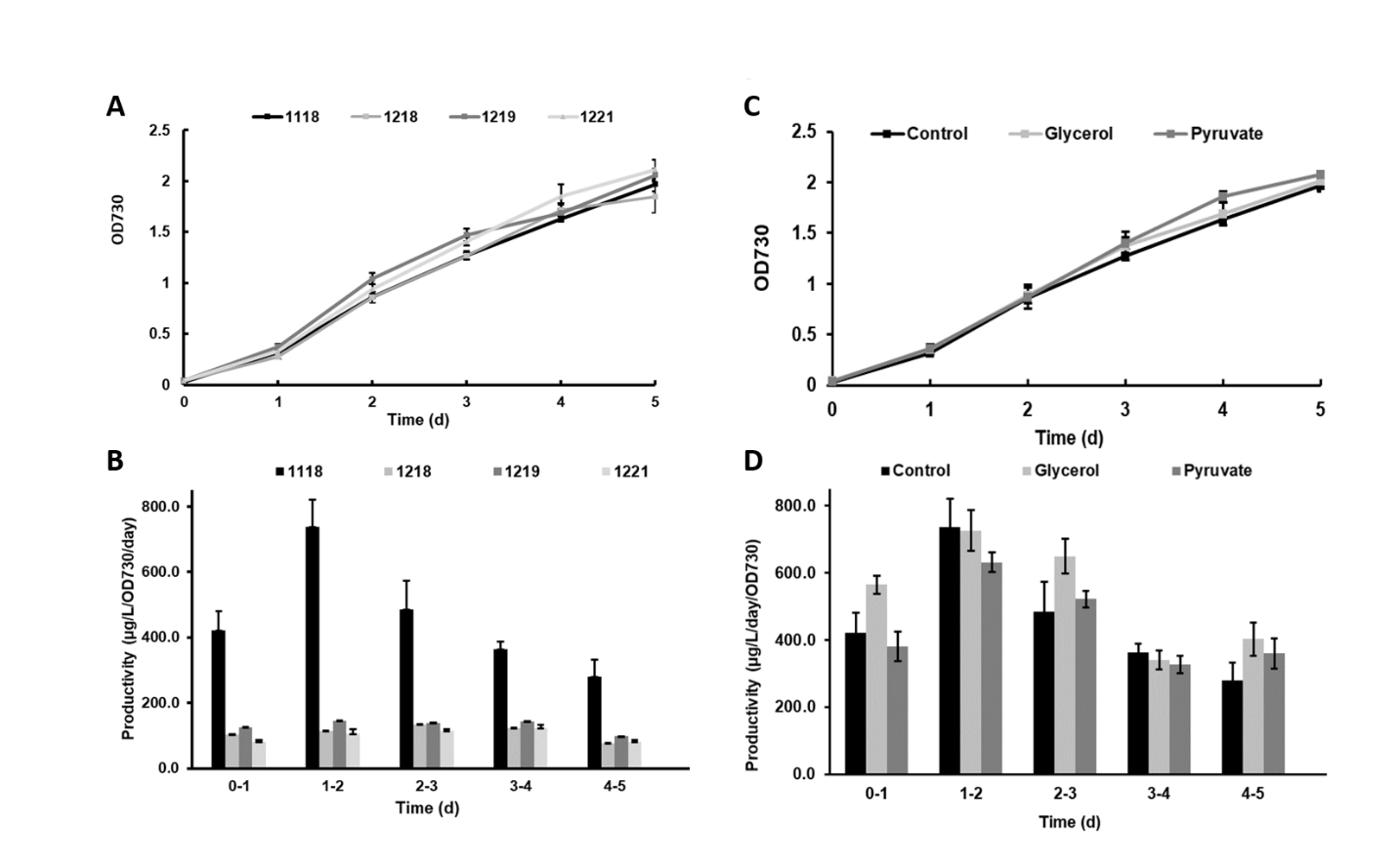


**Figure S2.** Effects of MEP pathway engineering and substrate feeding on cell growth and limonene production. A, growth of L1118 (control), L1218 (overexpression of *ispG* from *Synechocystis sp.* PCC 6803), L1219 (overexpression of *ispG* from *Botryococcus braunii*), and L1221 (overexpression of *rib* from *Escherichia coli* and and *ispG* from *Botryococcus braunii*). B, limonene specific productivity of L1118, L1218, L1219 and L1221. Growth and limonene specific productivities with glycerol and pyruvate feeding are shown in C and D, respectively.

**
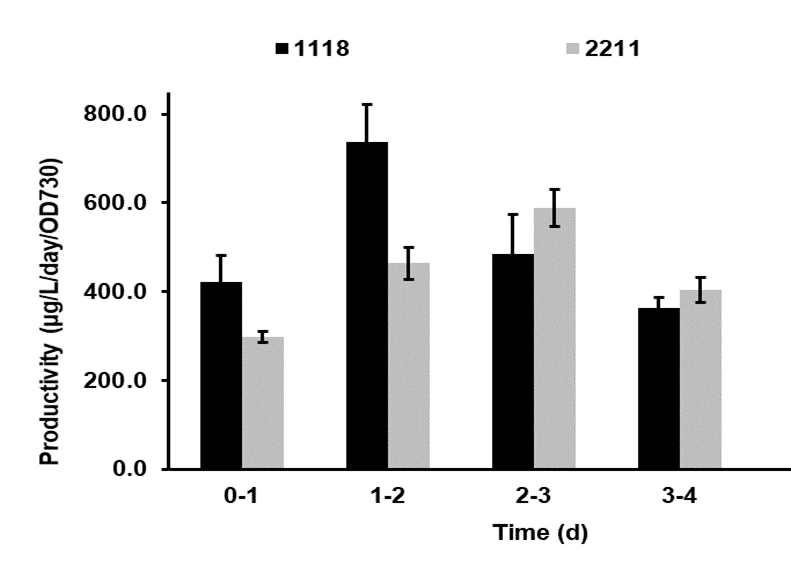
**

## **Figure S3.** Specific limonene productivity of engineered GAPDH and PGK strains. No increase was found in L2211 compared to L1118 in limonene productivity.


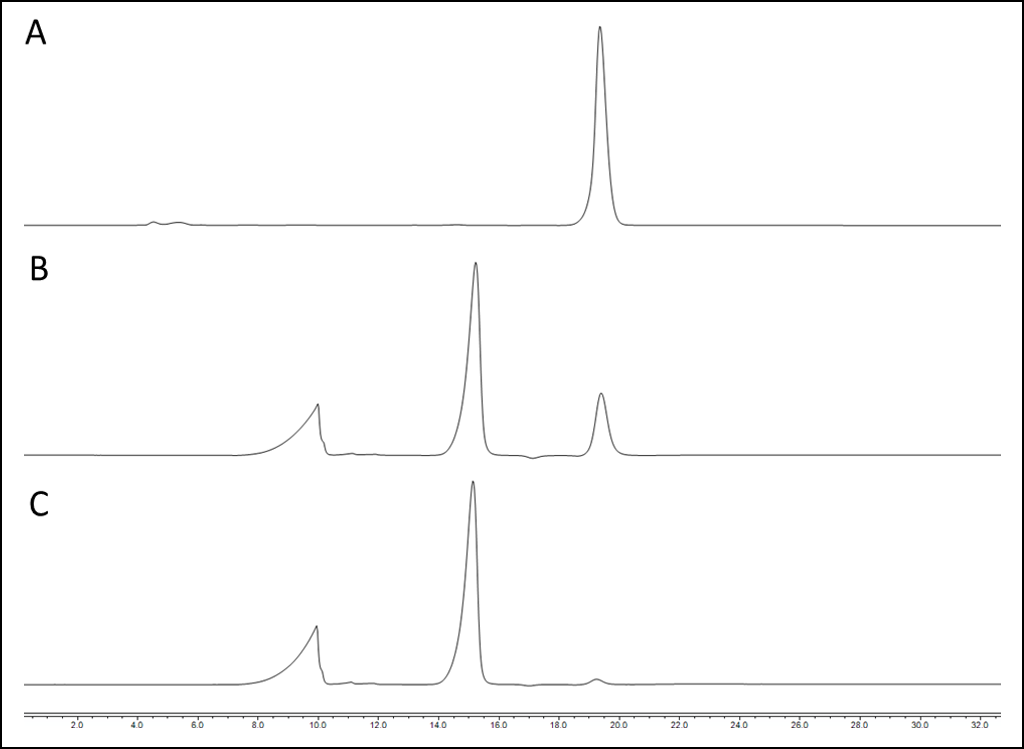


## **Figure S4.** Representative chromatogram of sucrose standard and chromatogram of L1118 and Lsps cell extracts for sucrose content measurement. A, Sucrose Standard B, 1118 Strain C, Lsps Strain. The sucrose retention time was 19.23 min and the peak was found at 10 g/L sucrose standard solution. The L1118 containing the corresponding peaks at RT 19.23 were collected and used for further calculation.


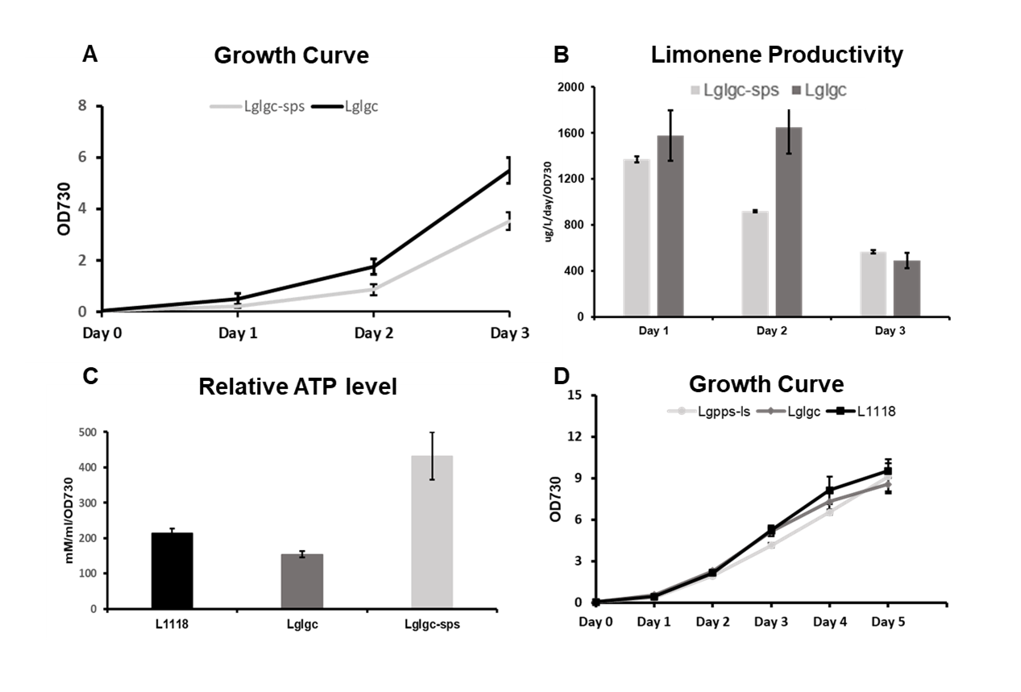


## **Figure S5**. Impacts of *glgC* and *sps* mutation. A, Slower growth in Lglgc-sps compared to Lglgc. B, Limonene productivity decrease in Lglgc-sps compared to Lglgc. C, Relative ATP level in L1118, Lglgc and Lglgc-sps. D, Growth comparison between L1118, Lglgc-ls and Lglgc.


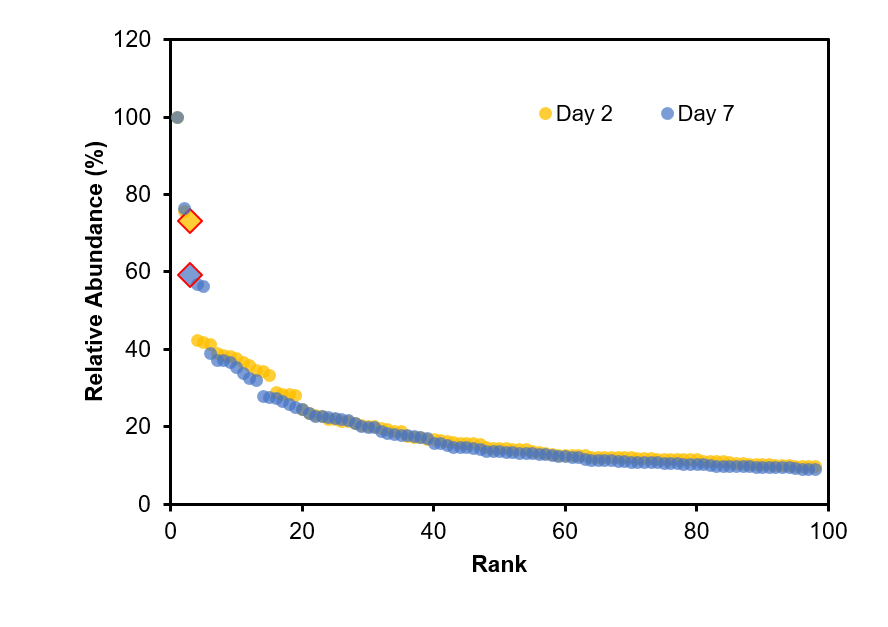


## **Figure S6**. Relative abundance of limonene synthase (LS) detected by proteomics. The LS (Diamonds with red borders) was found to be the third most abundant protein among all detected proteins either at day 2 or day 7. The relative abundance was calculated by normalizing NSAFs with the NSAF of the most abundant protein. The most abundant 100 proteins are shown in the figure.

# **Supplementary Table S1-S2**

**Table S1.** Strains and plasmids used in this study.

**Table S2.** Enzyme expression level changes in log phase and stationary phase in L1118.

**Table S3.** Proteomics analysis of strain Lsps comparing to L1118.

## **Table S1.** Strains and plasmids used in this study

| Strain or plasmid | Relevant characteristics | Source or reference |
| --- | --- | --- |
| Strains |  |  |
| *S. elongatus* PCC 7942 | Wild type | S. Golden |
| L1118 | *ls* at NS1 of *S. elongatus* PCC 7942 | X. Wang |
| Lsps | Δ*sps* of L1118 | This study |
| L1218 | *ispG* (from *Synechocystis* sp. PCC 6803) at NS2 of L1118 | This study |
| L1219 | *ispG* (from *Botryococcus braunii*) at NS2 of L1118 | This study |
| L1221 | *ribB* (Codon optimized for PCC 7942) and *ispG* (from *S. elongatus* PCC 7942) at NS2 of L1118 | This study |
| L2211 | *gap2* and *PGK* (from *Synechocystis* sp. PCC 6803) at NS2 of L1118 | This study |
| Plasmids |  |  |
| pAM2991 | Targeting PCC 7942 NS1; Ptrc; Sp/Sm^R^ | SS. Golden |
| pAM1579 | Targeting PCC 7942 NS2; P_L_lacO, Km^R^ | CR. Andersson |
| pLM21 | Targeting PCC 7942 *sps*, Gm^R^ | This study |
| pWX1218 | *ispG* (from *Synechocystis sp*. PCC 6803 in pAM1579, Km^R^ | This study |
| pWX1219 | *ispG* (from *Botryococcus braunii* in pAM1579, Km^R^ | This study |
| pWX1221 | *ribB* (Codon optimized for PCC 7942) and *ispG* (from *S. elongatus* PCC 7942 in pAM1579, Km^R^ | This study |
| pLM2211 | *gap2* and *PGK* (from *Synechocystis* sp. PCC 6803) in pAM1579, Km^R^ | This study |

## **Table S2.** Enzyme expression level changes in log phase and stationary phase in L1118.

| Metabolism | Enzymes | Fold Change Day 2 vs. Day 7 |
| --- | --- | --- |
|  | PS Ⅱ 12kDa extrinsic protein | 2.27 |
| Light reaction | NAD(P)H-QUINONE oxidoreductase subunits J, H, O | ~2 |
| CO_2_ assimilation & CBB cycle | CO2 hydration protein | 2.53 |
|  | Bicarbonate-binding protein CmpA | 2.89 |
|  | Fructose-1,6-bisphosphatase | 1.51 |
|  | Glyceraldehyde-3-phosphate dehydrogenase | 1.73 |
| Glycogen biosynthesis | 1,4-alpha-glucan branching enzyme GlgB | 2.61 |

## **Table S3.** Proteomics analysis of strain Lsps comparing to L1118.

| Metabolism | Proteins | Relative Abundance  Lsps vs. L1118 | p-value |
| --- | --- | --- | --- |
| Light reaction | NAD(P)H-quinone oxidoreductase subunits K, M, N | 0.33,0.29,0.47 | **,***,** |
|  | ATP synthase subunit beta | 0.36 | ** |
|  | ATPase | 0.23 | *** |
|  | Photosystem II reaction center CP47, CP43 | 0.40,0.39,0.44 | ***,*** |
|  | Photosystem II D2 protein | 0.36 | ** |
|  | Photosystem I reaction center subunit IV | 0.22 | *** |
| TCA Cycle | Isocitrate dehydrogenase [NADP] | 2.01 | ** |
| Sugar metabolism | Glucose-1-phosphate adenylyltransferase (AGPase) | 1.44 | ** |
|  | Phosphoglucomutase | 0.41 | ** |
| Fatty acid synthesis | 3-oxoacyl-(Acyl-carrier protein) reductase | 2.05 | *** |
|  | 3-oxoacyl-(acyl-carrier-protein] synthase | 0.19 | *** |

**: p-value<0.05, ***: p-value<0.01

# **Supplementary references**

1. Li X, Luo XX, Lu X, Duan JG, Xu GW. 2011. Metabolomics study of diabetic retinopathy using gas chromatography-mass spectrometry: a comparison of stages and subtypes diagnosed by Western and Chinese medicine. Molecular Biosystems 7:2228-2237.

2. Lee DY, Fiehn O. 2008. High quality metabolomic data for Chlamydomonas reinhardtii. Plant Methods 4.

# **Supplementary File S1-S5**

## **File S1:** Plasmid map and sequence of pLM21


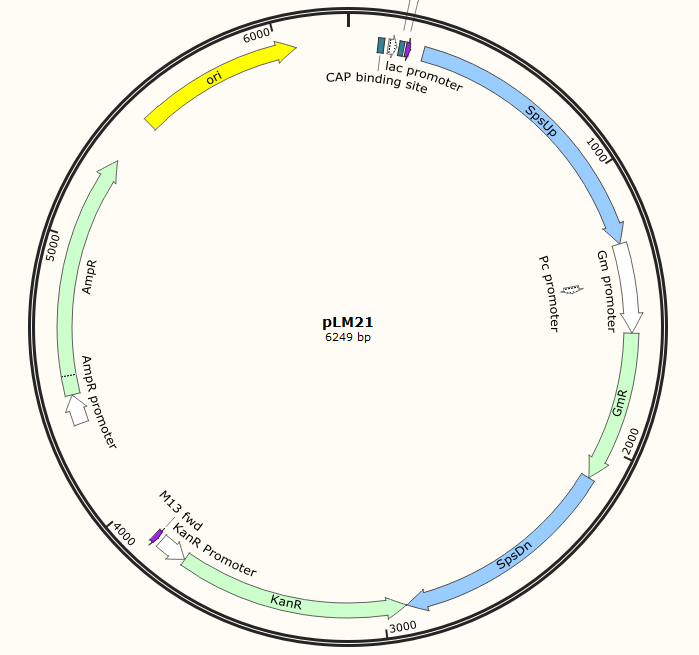


## Sequence:

GCGCCCAATACGCAAACCGCCTCTCCCCGCGCGTTGGCCGATTCATTAATGCAGCTGGCACGACAGGTTTCCCGACTGGAAAGCGGGCAGTGAGCGCAACGCAATTAATGTGAGTTAGCTCACTCATTAGGCACCCCAGGCTTTACACTTTATGCTTCCGGCTCGTATGTTGTGTGGAATTGTGAGCGGATAACAATTTCACACAGGAAACAGCTATGACCATGATTACGCCAAGCTTGCATGCCTGCAGGTCGACTCTAGAGGATCACAAGCTGTTTTACGCAATTTTTGCGACACTGCCCGATTTGGTGACGGAACTGGTGGAGGGTATTCCGCCCGAGGCAAAGTACCGGTTTTCGGCACCGGTGGTGAAGGCGCAAGAGTTTCGGCTAGATGGATTGCTGGAGCCGATCGAAGAAGGAAGCGGCTATCCAACAGTGTTTTTAGAAGCGCAGATGCAGAGCGATCGCGGCTTTTACAGTCGCTACTTTGCAGAGTTATTTGGTTACATCCGTCAGTATCCCGAGGCAGCCAATTGGAGAGGGCTATTGCTGATCAGGGCGCGATCGCTGGACTTAGGTGAAGAGTCGAGTTTTACGGAACTGCTGCAGGGACGAGTGCAGCGGCTGTATCTAGAGGATTTGATCGGGCGATCGCTGAATTCAGTGCGATTGCAGTTGCTGAAGCTATTGGTCGTACCAGTGGCAGCGATAGGAGAGACGGCGAGAGCGGTGTTGTTGTCAGCAGAGGATGAGGATGCGTTTGAGCAGCTACTCGAACTGGTGGAGGCTATAGTGGCAAGTAAGTTGCCTCAGCTTGAGATTGAGGAGATTCATCAAATGCTCGGTATTGAAGTCTCCGACTTAAAACAAACCCGCCTCTATCAAAGCGCGGTGGAAGAAGGTGAGAAAAAGGGACGCCAAGAAGGTGAGCTGGCTCTGGTCTTGAGGCAGCTGCAGCGGCGATTTGAGAACTTAACGGAAGAGCAGGAGCAGCAGATTCGATCGCTGTCATTGGAGGCGATCGAAGCCTTGAGTCTTGATTGGCTCGACTTTGAGACTGTCGCTGATTTAAATCGCTGGCTCCAAAAACATTAAATGCATGGGATTGCAAAAAAGACCTTAAGCCCACGCGCTTCTCTCAGCCCGTCGTGAAAAAGACTGCAATATCGCACTGAATACTTTGGCTGCCCGCTCAAGTGGAATGGTCAAGATTGGCAATCGCTACCTATTGCAGCAACGCGATCGCCCAATCTTTAACTGAAAAGGCGTGGAGACCGAAACCTTGCGCTCGTTCGCCAGCCAGGACAGAAATGCCTCGACTTCGCTGCTGCCCAAGGTTGCCGGGTGACGCACACCGTGGAAACGGATGAAGGCACGAACCCAGTTGACATAAGCCTGTTCGGTTCGTAAACTGTAATGCAAGTAGCGTATGCGCTCACGCAACTGGTCCAGAACCTTGACCGAACGCAGCGGTGGTAACGGCGCAGTGGCGGTTTTCATGGCTTGTTATGACTGTTTTTTTGTACAGTCTATGCCTCGGGCATCCAAGCAGCAAGCGCGTTACGCCGTGGGTCGATGTTTGATGTTATGGAGCAGCAACGATGTTACGCAGCAGGGCAGTCGCCCTAAAACAAAGTTAGGTGGCTCAAGTATGGGCATCATTCGCACATGTAGGCTCGGCCCTGACCAAGTCAAATCCATGCGGGCTGCTCTTGATCTTTTCGGTCGTGAGTTCGGAGACGTAGCCACCTACTCCCAACATCAGCCGGACTCCGATTACCTCGGGAACTTGCTCCGTAGTAAGACATTCATCGCGCTTGCTGCCTTCGACCAAGAAGCGGTTGTTGGCGCTCTCGCGGCTTACGTTCTGCCCAAGTTTGAGCAGCCGCGTAGTGAGATCTATATCTATGATCTCGCAGTCTCCGGCGAGCACCGGAGGCAGGGCATTGCCACCGCGCTCATCAATCTCCTCAAGCATGAGGCCAACGCGCTTGGTGCTTATGTGATCTACGTGCAAGCAGATTACGGTGACGATCCCGCAGTGGCTCTCTATACAAAGTTGGGCATACGGGAAGAAGTGATGCACTTTGATATCGACCCAAGTACCGCCACCTAAAAGCGGCCAGTCTTGCTCCAACAGCTTTTTTAGCTTGATCCGCCCCAGAGAATGCCCTGTGAAAATTAGCGGTACATTCAACCAGCGACTCAGCAGTGATCCCACTTGGCCAGCATCAGCATAGTGGGCCTGAATCCAAGTCGGGGTGCGCTTTTGCTGAGCCAGATATTGGAGAATTGCATCCGCAAAGGTGTAGAGATGGGGCCAAAGCAGCTCTTTACGGAGGTAGCGTTTGGGGCCAAAAGGCAAACGGACAATCCGACCTTTGGGCGCAAAGGGTTCGATCGCCTGACTGTAACCAACACTGACGCGGGGGTCGGTGATTTGGCGGGTGATGATGTCGACTTGTTGGACTTGTGGGGATTTAGCTTGGGCTTGAGCCAGTTCTAAGACGTACTTGGTCTGCCCGCCGGTGTCGGCATCTCGCCCCAGTTCCAAGTTCTGCCCTCGCAGCAGACCATGGGTCTGAATGTGCAGAATGTAGAGATTTTGAGCTGCCACGCGCTAGTCAGCCTCACAGAAAACGCCCCCATCCTAGTCTCCGATCCGAAAAGGTGGGGTGGTAGCGTAGGGGCGTTGCATGGCTGGTATCGATGTTTTCGAGCCGCTCACCAAGGATTATGGGCCCGCGCCGACCCGCGATCGCGATCGCCCTCGGTCTGCTGCTGTTAGCTTTCCTGATCTTGGTGGGGTTGAGTCTTGGCTCCTCAACAAGTCTGATGTCTCACGATGAGGGCTATTACGCCCTGCAGGCCCGTTGGATTGTGGAAACGGGTGATTGGGTAACGCCGCGCTGGTGGTTAGAAAAACTCATCGAGCATCAAATGAAACTGCAATTTATTCATATCAGGATTATCAATACCATATTTTTGAAAAAGCCGTTTCTGTAATGAAGGAGAAAACTCACCGAGGCAGTTCCATAGGATGGCAAGATCCTGGTATCGGTCTGCGATTCCGACTCGTCCAACATCAATACAACCTATTAATTTCCCCTCGTCAAAAATAAGGTTATCAAGTGAGAAATCACCATGAGTGACGACTGAATCCGGTGAGAATGGCAAAAGCTTATGCATTTCTTTCCAGACTTGTTCAACAGGCCAGCCATTACGCTCGTCATCAAAATCACTCGCATCAACCAAACCGTTATTCATTCGTGATTGCGCCTGAGCGAGACGAAATACGCGATCGCTGTTAAAAGGACAATTACAAACAGGAATCGAATGCAACCGGCGCAGGAACACTGCCAGCGCATCAACAATATTTTCACCTGAATCAGGATATTCTTCTAATACCTGGAATGCTGTTTTCCCGGGGATCGCAGTGGTGAGTAACCATGCATCATCAGGAGTACGGATAAAATGCTTGATGGTCGGAAGAGGCATAAATTCCGTCAGCCAGTTTAGTCTGACCATCTCATCTGTAACATCATTGGCAACGCTACCTTTGCCATGTTTCAGAAACAACTCTGGCGCATCGGGCTTCCCATACAATCGATAGATTGTCGCACCTGATTGCCCGACATTATCGCGAGCCCATTTATACCCATATAAATCAGCATCCATGTTGGAATTTAATCGCGGCCTCGAGCAAGACGTTTCCCGTTGAATATGGCTCATAACACCCCTTGTATTACTGTTTATGTAAGCAGACAGTTTTATTGTTCATGATGATATATTTTTATCTTGTGCAATGTAACATCAGAGATTTTGAGACACAACGTGGGAGCTCGAATTCACTGGCCGTCGTTTTACAACGTCGTGACTGGGAAAACCCTGGCGTTACCCAACTTAATCGCCTTGCAGCACATCCCCCTTTCGCCAGCTGGCGTAATAGCGAAGAGGCCCGCACCGATCGCCCTTCCCAACAGTTGCGCAGCCTGAATGGCGAATGGCGCCTGATGCGGTATTTTCTCCTTACGCATCTGTGCGGTATTTCACACCGCATATGGTGCACTCTCAGTACAATCTGCTCTGATGCCGCATAGTTAAGCCAGCCCCGACACCCGCCAACACCCGCTGACGCGCCCTGACGGGCTTGTCTGCTCCCGGCATCCGCTTACAGACAAGCTGTGACCGTCTCCGGGAGCTGCATGTGTCAGAGGTTTTCACCGTCATCACCGAAACGCGCGAGACGAAAGGGCCTCGTGATACGCCTATTTTTATAGGTTAATGTCATGATAATAATGGTTTCTTAGACGTCAGGTGGCACTTTTCGGGGAAATGTGCGCGGAACCCCTATTTGTTTATTTTTCTAAATACATTCAAATATGTATCCGCTCATGAGACAATAACCCTGATAAATGCTTCAATAATATTGAAAAAGGAAGAGTATGAGTATTCAACATTTCCGTGTCGCCCTTATTCCCTTTTTTGCGGCATTTTGCCTTCCTGTTTTTGCTCACCCAGAAACGCTGGTGAAAGTAAAAGATGCTGAAGATCAGTTGGGTGCACGAGTGGGTTACATCGAACTGGATCTCAACAGCGGTAAGATCCTTGAGAGTTTTCGCCCCGAAGAACGTTTTCCAATGATGAGCACTTTTAAAGTTCTGCTATGTGGCGCGGTATTATCCCGTATTGACGCCGGGCAAGAGCAACTCGGTCGCCGCATACACTATTCTCAGAATGACTTGGTTGAGTACTCACCAGTCACAGAAAAGCATCTTACGGATGGCATGACAGTAAGAGAATTATGCAGTGCTGCCATAACCATGAGTGATAACACTGCGGCCAACTTACTTCTGACAACGATCGGAGGACCGAAGGAGCTAACCGCTTTTTTGCACAACATGGGGGATCATGTAACTCGCCTTGATCGTTGGGAACCGGAGCTGAATGAAGCCATACCAAACGACGAGCGTGACACCACGATGCCTGTAGCAATGGCAACAACGTTGCGCAAACTATTAACTGGCGAACTACTTACTCTAGCTTCCCGGCAACAATTAATAGACTGGATGGAGGCGGATAAAGTTGCAGGACCACTTCTGCGCTCGGCCCTTCCGGCTGGCTGGTTTATTGCTGATAAATCTGGAGCCGGTGAGCGTGGGTCTCGCGGTATCATTGCAGCACTGGGGCCAGATGGTAAGCCCTCCCGTATCGTAGTTATCTACACGACGGGGAGTCAGGCAACTATGGATGAACGAAATAGACAGATCGCTGAGATAGGTGCCTCACTGATTAAGCATTGGTAACTGTCAGACCAAGTTTACTCATATATACTTTAGATTGATTTAAAACTTCATTTTTAATTTAAAAGGATCTAGGTGAAGATCCTTTTTGATAATCTCATGACCAAAATCCCTTAACGTGAGTTTTCGTTCCACTGAGCGTCAGACCCCGTAGAAAAGATCAAAGGATCTTCTTGAGATCCTTTTTTTCTGCGCGTAATCTGCTGCTTGCAAACAAAAAAACCACCGCTACCAGCGGTGGTTTGTTTGCCGGATCAAGAGCTACCAACTCTTTTTCCGAAGGTAACTGGCTTCAGCAGAGCGCAGATACCAAATACTGTTCTTCTAGTGTAGCCGTAGTTAGGCCACCACTTCAAGAACTCTGTAGCACCGCCTACATACCTCGCTCTGCTAATCCTGTTACCAGTGGCTGCTGCCAGTGGCGATAAGTCGTGTCTTACCGGGTTGGACTCAAGACGATAGTTACCGGATAAGGCGCAGCGGTCGGGCTGAACGGGGGGTTCGTGCACACAGCCCAGCTTGGAGCGAACGACCTACACCGAACTGAGATACCTACAGCGTGAGCTATGAGAAAGCGCCACGCTTCCCGAAGGGAGAAAGGCGGACAGGTATCCGGTAAGCGGCAGGGTCGGAACAGGAGAGCGCACGAGGGAGCTTCCAGGGGGAAACGCCTGGTATCTTTATAGTCCTGTCGGGTTTCGCCACCTCTGACTTGAGCGTCGATTTTTGTGATGCTCGTCAGGGGGGCGGAGCCTATGGAAAAACGCCAGCAACGCGGCCTTTTTACGGTTCCTGGCCTTTTGCTGGCCTTTTGCTCACATGTTCTTTCCTGCGTTATCCCCTGATTCTGTGGATAACCGTATTACCGCCTTTGAGTGAGCTGATACCGCTCGCCGCAGCCGAACGACCGAGCGCAGCGAGTCAGTGAGCGAGGAAGCGGAAGA

## **File S2:** Plasmid map and sequence of pWX1218


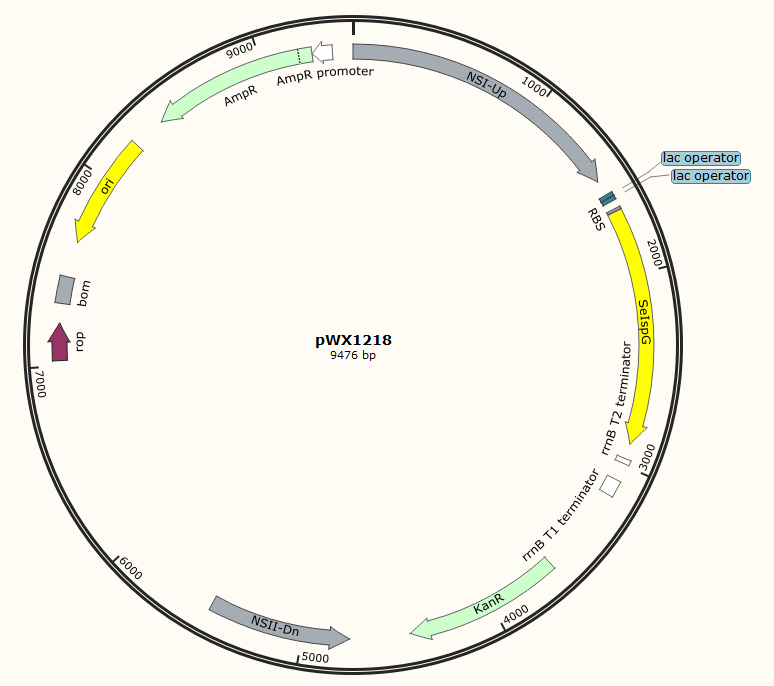


## Sequence:

AGCTTGTCATCTGCCGGATGAGGCAAAACCCTGCCTACGGCGCGATTACATCGTCCCAGCGCGATCGCTCTTACTGTTGATGGCTCGTGCTTAAAAACAATGCAAACTTCACCGTTTCAGCTGGTGATTTTCGACTGTGATGGTGTGCTTGTTGATAGCGAACGCATCACTAATCGCGTCTTTGCAGACATGCTCAATGAACTGGGTCTGTTGGTGACTTTGGATGACATGTTTGAGCAGTTTGTGGGTCATTCCATGGCTGACTGTCTCAAACTAATTGAGCGACGGTTAGGCAATCCTCCACCCCCTGACTTTGTTCAGCACTATCAACGCCGTACCCGTATCGCGTTAGAAACGCATCTACAAGCCGTTCCTGGGGTTGAAGAGGCTTTGGATGCTCTTGAATTGCCCTACTGTGTTGCGTCCAGTGGTGATCATCAAAAGATGCGAACCACACTGAGCCTGACGAAGCTCTGGCCACGATTTGAGGGACGAATCTTCAGCGTGACTGAAGTACCTCGCGGCAAGCCATTTCCCGATGTCTTTTTGTTGGCCGCCGATCGCTTCGGGGTTAATCCTACGGCCTGCGCTGTGATCGAAGACACCCCCTTGGGAGTAGCGGCAGGCGTGGCGGCAGGAATGCAAGTGTTTGGCTACGCGGGTTCCATGCCCGCTTGGCGTCTGCAAGAAGCCGGTGCCCATCTCATTTTTGACGATATGCGACTGCTGCCCAGTCTGCTCCAATCGTCGCCAAAAGATAACTCCACAGCATTGCCCAATCCCTAACCCCTGCTCGCGCCGCAACTACACACTAAACCGTTCCTGCGCGATCGCTCTTACTGTTGATGGCTCGTGCTTAAAAACAATGCAACCCTAACCGTTTCAGCTGGTGATTTTCGGACGATTTGGCTTACAGGGATAACTGAGAGTCAACAGCCTCTGTCCGTCATTGCACACCCATCCATGCACTGGGGACTTGACTCATGCTGAATCACATTTCCCTTGTCCATTGGGCGAGAGGGGAGGGGAATCTTCTGGACTCTTCACTAAGCGGCGATCGCAGGTTCTTCTACCCAAGCAGTGGCGATCGCTTGATTGCAGTCTTCAATGCTGGCCTCTGCAGCCATCGCCGCCACCAAAGCATCGTAGGCGGGACGTTGTTGCTCCAGTAAAGTCTTCGCCCGTAACAATCCCCAGCGACTGCGTAAATCCGCTTCGGCAGGATTGCGATCGAGTTGCCGCCACAGTTGTTTCCACTGGGCGCGATCGTCAGCTCCCCCTTCCACGTTGCCGTAGACCAGTTGCTCTGCCGCTGCACCGGCCATCAACACCTGACACCACTGTTCCAGCGATCGCTGACTGAGTTGCCCCTGTGCGGCTTCGGCTTCTAGCGCAGCTGCTTGGAACTGCACACCCCCGCGACCAGGTTGTCCTTGGCGCAGCGCTTCCCACGCTGAGAGGGTGTAGCCCGTCACGGGTAACCGATTAAGAAACCATTATTATCATGACATTAACCTATAAAAATAGGCGTATCACGAGGCCCTTTCGTCTTCACCTCGAGAATTGTGAGCGGATAACAATTGACATTGTGAGCGGATAACAAGATACTGAGCACATCAGCAGGACGCACTGACCGAATTCATTAAAGAGGAGAAAGATATCATGCAGACCCTCTCCACCCCCAGCACAACTGCCACTGAGTTTGATACCGTCATCCACCGGCGCCCTACGCGATCGGTTCGGGTCGGTGATATCTGGATTGGCAGCCGCCATCCCGTCGTTGTCCAGTCGATGATCAACGAGGACACCCTCGACATCGATGGGTCTGTTGCCGCGATCCGCCGCCTGCATGAGATTGGCTGCGAGATCGTCCGCGTCACGGTGCCCAGTCTTGGCCATGCCAAAGCGGTCGGTGACATTAAAAAGAAACTGCAAGACACCTATCGCGACGTGCCCTTGGTTGCCGACGTGCACCACAACGGTATGAAGATCGCGCTGGAAGTCGCCAAGCACGTTGACAAAGTGCGGATCAATCCTGGTCTCTACGTCTTTGAGAAGCCCGATCCGAATCGTCAGGGCTACACACCAGAAGAATTTGAGCGAATTGGCAAGCAAATTCGCGACACGCTTGAGCCCTTAGTCACCAGCCTGCGCGAGCAGGACAAGGCCATGCGGATTGGCGTCAACCACGGCTCTCTAGCCGAGCGGATGCTGTTCACCTACGGCGACACGCCGGAAGGCATGGTGGAATCGGCGTTGGAATTCCTGCGACTCTGCGAAGAGATGGACTTCCGTAATCTCGTCATCTCCATGAAAGCGAGCCGAGCGCCGGTGATGATGGCGGCCTATCGCCTGATGGCCAAACGGATGGATGATCTGGGCATGGATTATCCGCTCCACTTGGGTGTGACCGAAGCCGGTGATGGTGACTATGGCCGGATCAAATCGACGGTTGGGATTGGCACGCTGCTAGCGGAAGGGATTGGCGATACGATTCGCGTTTCGCTCACGGAAGCCCCCGAAAACGAAATTCCGGTTTGCTATTCGATTTTGCAAGCCCTCGGCCTCCGCAAAACCATGGTTGAGTATGTCGCCTGTCCAAGTTGCGGACGGACACTCTTCAATCTGGAAGAGGTCCTTCACAAAGTGCGGGCTGCAACCAATCATTTGGTTGGCTTGGATATTGCTGTCATGGGTTGCATCGTCAATGGCCCTGGCGAAATGGCTGATGCTGACTACGGCTACGTTGGCAAAACCCCAGGAACGATCGCCCTTTACCGAGGACGAGATGAAATCAAACGAGTTCCCGAAGAGCAAGGTGTAGAAGAACTGATCAACCTGATCAAAGCAGATGGACGCTGGGTTGAACCGGAACCGATTGCCTAAGTCGACAGGCCTCTAGACCCGGGCTCGAGCTAGCAAGCTTGGCCGGATCCGGCCGGATCCGGAGTTTGTAGAAACGCAAAAAGGCCATCCGTCAGGATGGCCTTCTGCTTAATTTGATGCCTGGCAGTTTATGGCGGGCGTCCTGCCCGCCACCCTCCGGGCCGTTGCTTCGCAACGTTCAAATCCGCTCCCGGCGGATTTGTCCTACTCAGGAGAGCGTTCACCGACAAACAACAGATAAAACGAAAGGCCCAGTCTTTCGACTGAGCCTTTCGTTTTATTTGATGCCTGGCAGTTCCCTACTCTCGCATGGGGAGACCCCACACTACCATCGGCGCTACGGCGTTTCACTTCTGAGTTCGGCATGGGGTCAGGTGGGACCACCGCGCTACTGCCGCCAGGCAAATTCTGTTTTATCAGCCGTTACCCCACCTACTAGCTAATCCCATCTGGGCACATCCGATGGCAAGAGGCCCGAAGGTCCCCCTCTTTGGTCTTGCGACGTTATGCGGTATTAGCTACCGTTTCCAGTAGTTATCCCCCTCCATCAGGCAGTTTCCCAGACATTACTCACCCGTCCGCCACTCGTCAGCAAAGAAGCAAGCTTAGATCGACCTGCAGGGGGGGGGGGGAAAGCCACGTTGTGTCTCAAAATCTCTGATGTTACATTGCACAAGATAAAAATATATCATCATGAACAATAAAACTGTCTGCTTACATAAACAGTAATACAAGGGGTGTTATGAGCCATATTCAACGGGAAACGTCTTGCTCGAGGCCGCGATTAAATTCCAACATGGATGCTGATTTATATGGGTATAAATGGGCTCGCGATAATGTCGGGCAATCAGGTGCGACAATCTATCGATTGTATGGGAAGCCCGATGCGCCAGAGTTGTTTCTGAAACATGGCAAAGGTAGCGTTGCCAATGATGTTACAGATGAGATGGTCAGACTAAACTGGCTGACGGAATTTATGCCTCTTCCGACCATCAAGCATTTTATCCGTACTCCTGATGATGCATGGTTACTCACCACTGCGATCCCCGGGAAAACAGCATTCCAGGTATTAGAAGAATATCCTGATTCAGGTGAAAATATTGTTGATGCGCTGGCAGTGTTCCTGCGCCGGTTGCATTCGATTCCTGTTTGTAATTGTCCTTTTAACAGCGATCGCGTATTTCGTCTCGCTCAGGCGCAATCACGAATGAATAACGGTTTGGTTGATGCGAGTGATTTTGATGACGAGCGTAATGGCTGGCCTGTTGAACAAGTCTGGAAAGAAATGCATAAGCTTTTGCCATTCTCACCGGATTCAGTCGTCACTCATGGTGATTTCTCACTTGATAACCTTATTTTTGACGAGGGGAAATTAATAGGTTGTATTGATGTTGGACGAGTCGGAATCGCAGACCGATACCAGGATCTTGCCATCCTATGGAACTGCCTCGGTGAGTTTTCTCCTTCATTACAGAAACGGCTTTTTCAAAAATATGGTATTGATAATCCTGATATGAATAAATTGCAGTTTCATTTGATGCTCGATGAGTTTTTCTAATCAGAATTGGTTAATTGGTTGTAACACTGGCAGAGCATTACGCTGACTTGACGGGACGGCGGCTTTGTTGAATAAATCGAACTTTTGCTGAGTTGAAGGATCAGATCACGCATCTTCCCGACAACGCAGACCGTTCCGTGGCAAAGCAAAAGTTCAAAATCACCAACTGGTCCACCTACAACAAAGCTCTCATCAACCGTGGCTCCCTCACTTTCTGGCTGGATGATGGGGCGATTCAGGCCTGGTATGAGTCAGCAACACCTTCTTCACGAGGCAGACCTCAGCGCCCCCCCCCCCCTGCAGGTCGATCTGGTAACCCCAGCGCGGTTGCTACCAAGTAGTGACCCGCTTCGTGATGCAAAATCCGCTGACGATATTCGGGCGATCGCTGCTGAATGCCATCGAGCAGTAACGTGGCACCCCGCCCCTGCCAAGTCACCGCATCCAGACTGAACAGCACCAAGAGGCTAAAACCCAATCCCGCCGGTAGCAGCGGAGAACTACCCAGCATTGGTCCCACCAAAGCTAATGCCGTCGTGGTAAAAATCGCGATCGCCGTCAGACTCAAGCCCAGTTCGCTCATGCTTCCTCATCTAGGTCACAGTCTTCGGCGATCGCATCGATCTGATGCTGCAGCAAGCGTTTTCCATACCGGCGATCGCGCCGTCGCCCTTTCGCTGCCGTGGCCCGCTTACGAGCTCGTTTATCGACCACGATCGCATCCAAATCCGCGATCGCTTCCCAGTCCGGCAATTCAGTCTGGGGCGTCCGTTTCATTAATCCTGATCAGGCACGAAATTGCTGTGCGTAGTATCGCGCATAGCGGCCAGCCTCTGCCAACAGCGCATCGTGATTGCCTGCCTCAACAATCTGGCCGCGCTCCATCACCAAGATGCGGCTGGCATTACGAACCGTAGCCAGACGGTGAGCAATGATAAAGACCGTCCGTCCCTGCATCACCCGTTCTAGGGCCTCTTGCACCAAGGTTTCGGACTCGGAATCAAGCGCCGAAGTCGCCTCATCCAGAATTAAAATGCGTGGATCCTCTACGCCGGACGCATCGTGGCCGGCATCACCGGCGCCACAGGTGCGGTTGCTGGCGCCTATATCGCCGACATCACCGATGGGGAAGATCGGGCTCGCCACTTCGGGCTCATGAGCGCTTGTTTCGGCGTGGGTATGGTGGCAGGCCCCGTGGCCGGGGGACTGTTGGGCGCCATCTCCTTGCATGCACCATTCCTTGCGGCGGCGGTGCTCAACGGCCTCAACCTACTACTGGGCTGCTTCCTAATGCAGGAGTCGCATAAGGGAGAGCGTCGATCGACCGATGCCCTTGAGAGCCTTCAACCCAGTCAGCTCCTTCCGGTGGGCGCGGGGCATGACTATCGTCGCCGCACTTATGACTGTCTTCTTTATCATGCAACTCGTAGGACAGGTGCCGGCAGCGCTCTGGGTCATTTTCGGCGAGGACCGCTTTCGCTGGAGCGCGACGATGATCGGCCTGTCGCTTGCGGTATTCGGAATCTTGCACGCCCTCGCTCAAGCCTTCGTCACTGGTCCCGCCACCAAACGTTTCGGCGAGAAGCAGGCCATTATCGCCGGCATGGCGGCCGACGCGCTGGGCTACGTCTTGCTGGCGTTCGCGACGCGAGGCTGGATGGCCTTCCCCATTATGATTCTTCTCGCTTCCGGCGGCATCGGGATGCCCGCGTTGCAGGCCATGCTGTCCAGGCAGGTAGATGACGACCATCAGGGACAGCTTCAAGGATCGCTCGCGGCTCTTACCAGCCTAACTTCGATCACTGGACCGCTGATCGTCACGGCGATTTATGCCGCCTCGGCGAGCACATGGAACGGGTTGGCATGGATTGTAGGCGCCGCCCTATACCTTGTCTGCCTCCCCGCGTTGCGTCGCGGTGCATGGAGCCGGGCCACCTCGACCTGAATGGAAGCCGGCGGCACCTCGCTAACGGATTCACCACTCCAAGAATTGGAGCCAATCAATTCTTGCGGAGAACTGTGAATGCGCAAACCAACCCTTGGCAGAACATATCCATCGCGTCCGCCATCTCCAGCAGCCGCACGCGGCGCATCTCGGGCAGCGTTGGGTCCTGGCCACGGGTGCGCATGATCGTGCTCCTGTCGTTGAGGACCCGGCTAGGCTGGCGGGGTTGCCTTACTGGTTAGCAGAATGAATCACCGATACGCGAGCGAACGTGAAGCGACTGCTGCTGCAAAACGTCTGCGACCTGAGCAACAACATGAATGGTCTTCGGTTTCCGTGTTTCGTAAAGTCTGGAAACGCGGAAGTCAGCGCCCTGCACCATTATGTTCCGGATCTGCATCGCAGGATGCTGCTGGCTACCCTGTGGAACACCTACATCTGTATTAACGAAGCGCTGGCATTGACCCTGAGTGATTTTTCTCTGGTCCCGCCGCATCCATACCGCCAGTTGTTTACCCTCACAACGTTCCAGTAACCGGGCATGTTCATCATCAGTAACCCGTATCGTGAGCATCCTCTCTCGTTTCATCGGTATCATTACCCCCATGAACAGAAATCCCCCTTACACGGAGGCATCAGTGACCAAACAGGAAAAAACCGCCCTTAACATGGCCCGCTTTATCAGAAGCCAGACATTAACGCTTCTGGAGAAACTCAACGAGCTGGACGCGGATGAACAGGCAGACATCTGTGAATCGCTTCACGACCACGCTGATGAGCTTTACCGCAGCTGCCTCGCGCGTTTCGGTGATGACGGTGAAAACCTCTGACACATGCAGCTCCCGGAGACGGTCACAGCTTGTCTGTAAGCGGATGCCGGGAGCAGACAAGCCCGTCAGGGCGCGTCAGCGGGTGTTGGCGGGTGTCGGGGCGCAGCCATGACCCAGTCACGTAGCGATAGCGGAGTGTATACTGGCTTAACTATGCGGCATCAGAGCAGATTGTACTGAGAGTGCACCATATGCGGTGTGAAATACCGCACAGATGCGTAAGGAGAAAATACCGCATCAGGCGCTCTTCCGCTTCCTCGCTCACTGACTCGCTGCGCTCGGTCGTTCGGCTGCGGCGAGCGGTATCAGCTCACTCAAAGGCGGTAATACGGTTATCCACAGAATCAGGGGATAACGCAGGAAAGAACATGTGAGCAAAAGGCCAGCAAAAGGCCAGGAACCGTAAAAAGGCCGCGTTGCTGGCGTTTTTCCATAGGCTCCGCCCCCCTGACGAGCATCACAAAAATCGACGCTCAAGTCAGAGGTGGCGAAACCCGACAGGACTATAAAGATACCAGGCGTTTCCCCCTGGAAGCTCCCTCGTGCGCTCTCCTGTTCCGACCCTGCCGCTTACCGGATACCTGTCCGCCTTTCTCCCTTCGGGAAGCGTGGCGCTTTCTCATAGCTCACGCTGTAGGTATCTCAGTTCGGTGTAGGTCGTTCGCTCCAAGCTGGGCTGTGTGCACGAACCCCCCGTTCAGCCCGACCGCTGCGCCTTATCCGGTAACTATCGTCTTGAGTCCAACCCGGTAAGACACGACTTATCGCCACTGGCAGCAGCCACTGGTAACAGGATTAGCAGAGCGAGGTATGTAGGCGGTGCTACAGAGTTCTTGAAGTGGTGGCCTAACTACGGCTACACTAGAAGGACAGTATTTGGTATCTGCGCTCTGCTGAAGCCAGTTACCTTCGGAAAAAGAGTTGGTAGCTCTTGATCCGGCAAACAAACCACCGCTGGTAGCGGTGGTTTTTTTGTTTGCAAGCAGCAGATTACGCGCAGAAAAAAAGGATCTCAAGAAGATCCTTTGATCTTTTCTACGGGGTCTGACGCTCAGTGGAACGAAAACTCACGTTAAGGGATTTTGGTCATGAGATTATCAAAAAGGATCTTCACCTAGATCCTTTTAAATTAAAAATGAAGTTTTAAATCAATCTAAAGTATATATGAGTAAACTTGGTCTGACAGTTACCAATGCTTAATCAGTGAGGCACCTATCTCAGCGATCTGTCTATTTCGTTCATCCATAGTTGCCTGACTCCCCGTCGTGTAGATAACTACGATACGGGAGGGCTTACCATCTGGCCCCAGTGCTGCAATGATACCGCGAGACCCACGCTCACCGGCTCCAGATTTATCAGCAATAAACCAGCCAGCCGGAAGGGCCGAGCGCAGAAGTGGTCCTGCAACTTTATCCGCCTCCATCCAGTCTATTAATTGTTGCCGGGAAGCTAGAGTAAGTAGTTCGCCAGTTAATAGTTTGCGCAACGTTGTTGCCATTGCTGCAGGCATCGTGGTGTCACGCTCGTCGTTTGGTATGGCTTCATTCAGCTCCGGTTCCCAACGATCAAGGCGAGTTACATGATCCCCCATGTTGTGCAAAAAAGCGGTTAGCTCCTTCGGTCCTCCGATCGTTGTCAGAAGTAAGTTGGCCGCAGTGTTATCACTCATGGTTATGGCAGCACTGCATAATTCTCTTACTGTCATGCCATCCGTAAGATGCTTTTCTGTGACTGGTGAGTACTCAACCAAGTCATTCTGAGAATAGTGTATGCGGCGACCGAGTTGCTCTTGCCCGGCGTCAACACGGGATAATACCGCGCCACATAGCAGAACTTTAAAAGTGCTCATCATTGGAAAACGTTCTTCGGGGCGAAAACTCTCAAGGATCTTACCGCTGTTGAGATCCAGTTCGATGTAACCCACTCGTGCACCCAACTGATCTTCAGCATCTTTTACTTTCACCAGCGTTTCTGGGTGAGCAAAAACAGGAAGGCAAAATGCCGCAAAAAAGGGAATAAGGGCGACACGGAAATGTTGAATACTCATACTCTTCCTTTTTCAATATTATTGAAGCATTTATCAGGGTTATTGTCTCATGAGCGGATACATATTTGAATGTATTTAGAAAAATAAACAAATAGGGGTTCCGCGCACATTTCCCCGAAAAGTGCCACCTGACGTCTAAGAAACCATTATTATCATGACATTAACCTATAAAAATAGGCGTATCACGAGGCCCTTTCGTCTTCAAGAATT

## **File S3:** Plasmid map and sequence of pWX1219


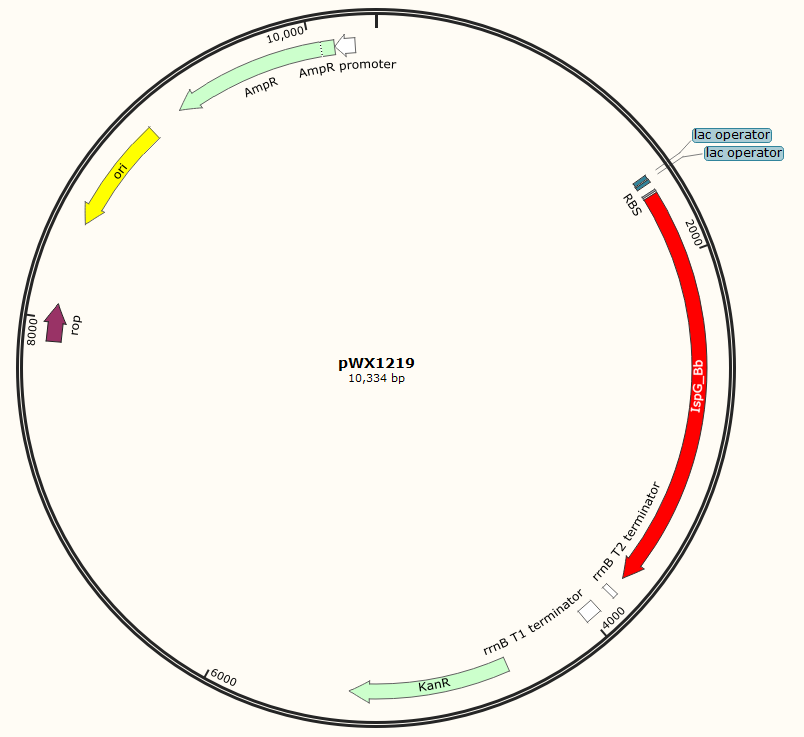


## Sequence:

AGCTTGTCATCTGCCGGATGAGGCAAAACCCTGCCTACGGCGCGATTACATCGTCCCAGCGCGATCGCTCTTACTGTTGATGGCTCGTGCTTAAAAACAATGCAAACTTCACCGTTTCAGCTGGTGATTTTCGACTGTGATGGTGTGCTTGTTGATAGCGAACGCATCACTAATCGCGTCTTTGCAGACATGCTCAATGAACTGGGTCTGTTGGTGACTTTGGATGACATGTTTGAGCAGTTTGTGGGTCATTCCATGGCTGACTGTCTCAAACTAATTGAGCGACGGTTAGGCAATCCTCCACCCCCTGACTTTGTTCAGCACTATCAACGCCGTACCCGTATCGCGTTAGAAACGCATCTACAAGCCGTTCCTGGGGTTGAAGAGGCTTTGGATGCTCTTGAATTGCCCTACTGTGTTGCGTCCAGTGGTGATCATCAAAAGATGCGAACCACACTGAGCCTGACGAAGCTCTGGCCACGATTTGAGGGACGAATCTTCAGCGTGACTGAAGTACCTCGCGGCAAGCCATTTCCCGATGTCTTTTTGTTGGCCGCCGATCGCTTCGGGGTTAATCCTACGGCCTGCGCTGTGATCGAAGACACCCCCTTGGGAGTAGCGGCAGGCGTGGCGGCAGGAATGCAAGTGTTTGGCTACGCGGGTTCCATGCCCGCTTGGCGTCTGCAAGAAGCCGGTGCCCATCTCATTTTTGACGATATGCGACTGCTGCCCAGTCTGCTCCAATCGTCGCCAAAAGATAACTCCACAGCATTGCCCAATCCCTAACCCCTGCTCGCGCCGCAACTACACACTAAACCGTTCCTGCGCGATCGCTCTTACTGTTGATGGCTCGTGCTTAAAAACAATGCAACCCTAACCGTTTCAGCTGGTGATTTTCGGACGATTTGGCTTACAGGGATAACTGAGAGTCAACAGCCTCTGTCCGTCATTGCACACCCATCCATGCACTGGGGACTTGACTCATGCTGAATCACATTTCCCTTGTCCATTGGGCGAGAGGGGAGGGGAATCTTCTGGACTCTTCACTAAGCGGCGATCGCAGGTTCTTCTACCCAAGCAGTGGCGATCGCTTGATTGCAGTCTTCAATGCTGGCCTCTGCAGCCATCGCCGCCACCAAAGCATCGTAGGCGGGACGTTGTTGCTCCAGTAAAGTCTTCGCCCGTAACAATCCCCAGCGACTGCGTAAATCCGCTTCGGCAGGATTGCGATCGAGTTGCCGCCACAGTTGTTTCCACTGGGCGCGATCGTCAGCTCCCCCTTCCACGTTGCCGTAGACCAGTTGCTCTGCCGCTGCACCGGCCATCAACACCTGACACCACTGTTCCAGCGATCGCTGACTGAGTTGCCCCTGTGCGGCTTCGGCTTCTAGCGCAGCTGCTTGGAACTGCACACCCCCGCGACCAGGTTGTCCTTGGCGCAGCGCTTCCCACGCTGAGAGGGTGTAGCCCGTCACGGGTAACCGATTAAGAAACCATTATTATCATGACATTAACCTATAAAAATAGGCGTATCACGAGGCCCTTTCGTCTTCACCTCGAGAATTGTGAGCGGATAACAATTGACATTGTGAGCGGATAACAAGATACTGAGCACATCAGCAGGACGCACTGACCGAATTCATTAAAGAGGAGAAAgatatcATGGCTACTGCCACACAAGATCCCACACTTGTTCTTGATCCATCTAAGATTGTGCTGCCTAAATATTGCGAGAGCACCTACAAGACTGTCCGGCGGCCAACACGGACGATCTATATTGGCAAAGTCCCAGTAGGATCGGAGCACCCTATCCGCTTGCAGACAATGACCACAACAGACACCAGGAATGTGGAAGCAACTGTTGATCAAGTAAAGAAGTGTGTCGATGTCGGTGCGGACTTCGTGCGCATCACCGTGCAGGGCAAGAAGGAGGCAGATGCTTGCATGCAGATCAGGGAGCGCCTCTTCAAAGACAGGTATGACGTACCTCTTGTCGCAGACATCCACTTCCAGCCCAAGGTTGCCCTAGCTGTTGCAGAAGCCTTCGAGAAGATCCGCATCAACCCAGGCAATTTTGCCGATGGTCGGAAGTCGTTCGAGGTCATCAACTACGATGATCCTGCCCAATTCACCCGAGAGCAGGAGGAAATCCGTGAGCTGTTCACCCCGCTAGTGGAGAAGTGCAAGTCTTTGAACCGTGCTATCCGTATTGGGACCAACCATGGCTCTCTGTCTGCCCGCATCCTGTCGTACTATGGGGACACCCCCAGAGGAATGGTAAATTCTGCCTTCGAATTTGCTGAGATTTGCCGGGACTTGGACTTCCACAACTTCTTGTTCAGCATGAAGGCCAGCAACCCTCTGGTCATGGTCCAGGCATACCGCCTCCTCGCTGAGGAAATGTACAACAAGGGCTGGGACTACCCACTGCATCTGGGTGTTACTGAGGCTGGTGAAGGAGAGGATGGCCGAATGAAGTCTGCCATTGGTATTGGATCTCTTTTAATGGATGGCCTGGGTGACACAATCCGTGTCTCCCTCACTGAGGACCCTGAGTTTGAGATTGTTCCTTGCGGGCAATTGGCCAACTTTGGGGCGCGGGCAGCCAAAGAGAACTGGGGTGTTGCGCCCTTCACAGAGGTCTACAGGGACACACACTCTTTCGCCCGCCGCACAGGCCGCCTGCCAGAGCAGCGGGAAGGTGACCAGTTGGATTTCCGCTCCCTGCTGCATCGCGATGGATCCGTGTTGTCCTTCGTACCTCTGGAGGAGCTGAAGCAGCCAGAGCTCTTGTACCGCAAACTGGGAGCCAAGCTTGTGGTTGGCATGCCTTTCAAGGACCTGGCTACGTCTGACACTTTGATGCTGCCTGAGGTGCCGCCATCTTCCGATGTGGAGGGCCGCCGCGCTCTGGCCCGCCTCCAGGAGGTAGGAGTGCATGTTGTCGCACCGCTGGCAGCCTTGGCAAAGGACCCACTGCCCAACGCAGTGGCATTGGTCTCCCTTCGGGATTATGCCGCAAAGGGAGTGACCCTCCCGGAGGGTGCTGCTAGGTTTGCCATCACAGCGGATGGTACTGAATCGGACGCTGAGGTGGAAGCCTTGAAGGACTCCAAAGCAGTCATGGTGCTGCTGGACGTGAAGAAGGGTGTCAGTCGGGTGCATGCTTCTCGCCACTTCTTCGAGTTGTTGCGGTTCTATGACATTGACCTCCCTGTCATCCACACTCGCCGTTTCCCTGCAGGATTGACCAGGGATGAGATTGTGCTGACATGCGGAGCGGAGATTGGAGCCCTGTTGGTGGATGGCCTCGGCGATGGTGTTCTATTGGACTGCCCCACAGAGGACCTAGACTTCATTCGGACAATGTCTTTCGGTCTCCTGCAGGGCTCACGCATGAGGAACACAAAGACAGAGTACGTATCATGCCCAAGCTGCGGGCGCACGCTGTTCGACCTCCAGGAGGTGACTGAGCAGATCAGGGTACGCACCGGCCACCTGCCAGGTGTTTCCATCGCCATTATGGGCTGCATTGTCAACGGCCCTGGTGAAATGGCGGATGCTGACTTCGGCTATGTAGGAGGCCGCCCCGGCCTCATCGACCTGTATGTTGGCAAAGAGGTTGTGAGGAAGAATATCCCCATGGAAGATGCCACCAATCAGCTTATTGAGCTCATCAAGGAATATGGCCGCTGGGTGGACAAGGAGGAGGAGGAGGTTGCTCAAGAACTGCAGGTTGCATAAGTCGACAGGCCTCTAGACCCGGGCTCGAGCTAGCAAGCTTGGCCGGATCCGGCCGGATCCGGAGTTTGTAGAAACGCAAAAAGGCCATCCGTCAGGATGGCCTTCTGCTTAATTTGATGCCTGGCAGTTTATGGCGGGCGTCCTGCCCGCCACCCTCCGGGCCGTTGCTTCGCAACGTTCAAATCCGCTCCCGGCGGATTTGTCCTACTCAGGAGAGCGTTCACCGACAAACAACAGATAAAACGAAAGGCCCAGTCTTTCGACTGAGCCTTTCGTTTTATTTGATGCCTGGCAGTTCCCTACTCTCGCATGGGGAGACCCCACACTACCATCGGCGCTACGGCGTTTCACTTCTGAGTTCGGCATGGGGTCAGGTGGGACCACCGCGCTACTGCCGCCAGGCAAATTCTGTTTTATCAGCCGTTACCCCACCTACTAGCTAATCCCATCTGGGCACATCCGATGGCAAGAGGCCCGAAGGTCCCCCTCTTTGGTCTTGCGACGTTATGCGGTATTAGCTACCGTTTCCAGTAGTTATCCCCCTCCATCAGGCAGTTTCCCAGACATTACTCACCCGTCCGCCACTCGTCAGCAAAGAAGCAAGCTTAGATCGACCTGCAGGGGGGGGGGGGAAAGCCACGTTGTGTCTCAAAATCTCTGATGTTACATTGCACAAGATAAAAATATATCATCATGAACAATAAAACTGTCTGCTTACATAAACAGTAATACAAGGGGTGTTATGAGCCATATTCAACGGGAAACGTCTTGCTCGAGGCCGCGATTAAATTCCAACATGGATGCTGATTTATATGGGTATAAATGGGCTCGCGATAATGTCGGGCAATCAGGTGCGACAATCTATCGATTGTATGGGAAGCCCGATGCGCCAGAGTTGTTTCTGAAACATGGCAAAGGTAGCGTTGCCAATGATGTTACAGATGAGATGGTCAGACTAAACTGGCTGACGGAATTTATGCCTCTTCCGACCATCAAGCATTTTATCCGTACTCCTGATGATGCATGGTTACTCACCACTGCGATCCCCGGGAAAACAGCATTCCAGGTATTAGAAGAATATCCTGATTCAGGTGAAAATATTGTTGATGCGCTGGCAGTGTTCCTGCGCCGGTTGCATTCGATTCCTGTTTGTAATTGTCCTTTTAACAGCGATCGCGTATTTCGTCTCGCTCAGGCGCAATCACGAATGAATAACGGTTTGGTTGATGCGAGTGATTTTGATGACGAGCGTAATGGCTGGCCTGTTGAACAAGTCTGGAAAGAAATGCATAAGCTTTTGCCATTCTCACCGGATTCAGTCGTCACTCATGGTGATTTCTCACTTGATAACCTTATTTTTGACGAGGGGAAATTAATAGGTTGTATTGATGTTGGACGAGTCGGAATCGCAGACCGATACCAGGATCTTGCCATCCTATGGAACTGCCTCGGTGAGTTTTCTCCTTCATTACAGAAACGGCTTTTTCAAAAATATGGTATTGATAATCCTGATATGAATAAATTGCAGTTTCATTTGATGCTCGATGAGTTTTTCTAATCAGAATTGGTTAATTGGTTGTAACACTGGCAGAGCATTACGCTGACTTGACGGGACGGCGGCTTTGTTGAATAAATCGAACTTTTGCTGAGTTGAAGGATCAGATCACGCATCTTCCCGACAACGCAGACCGTTCCGTGGCAAAGCAAAAGTTCAAAATCACCAACTGGTCCACCTACAACAAAGCTCTCATCAACCGTGGCTCCCTCACTTTCTGGCTGGATGATGGGGCGATTCAGGCCTGGTATGAGTCAGCAACACCTTCTTCACGAGGCAGACCTCAGCGCCCCCCCCCCCCTGCAGGTCGATCTGGTAACCCCAGCGCGGTTGCTACCAAGTAGTGACCCGCTTCGTGATGCAAAATCCGCTGACGATATTCGGGCGATCGCTGCTGAATGCCATCGAGCAGTAACGTGGCACCCCGCCCCTGCCAAGTCACCGCATCCAGACTGAACAGCACCAAGAGGCTAAAACCCAATCCCGCCGGTAGCAGCGGAGAACTACCCAGCATTGGTCCCACCAAAGCTAATGCCGTCGTGGTAAAAATCGCGATCGCCGTCAGACTCAAGCCCAGTTCGCTCATGCTTCCTCATCTAGGTCACAGTCTTCGGCGATCGCATCGATCTGATGCTGCAGCAAGCGTTTTCCATACCGGCGATCGCGCCGTCGCCCTTTCGCTGCCGTGGCCCGCTTACGAGCTCGTTTATCGACCACGATCGCATCCAAATCCGCGATCGCTTCCCAGTCCGGCAATTCAGTCTGGGGCGTCCGTTTCATTAATCCTGATCAGGCACGAAATTGCTGTGCGTAGTATCGCGCATAGCGGCCAGCCTCTGCCAACAGCGCATCGTGATTGCCTGCCTCAACAATCTGGCCGCGCTCCATCACCAAGATGCGGCTGGCATTACGAACCGTAGCCAGACGGTGAGCAATGATAAAGACCGTCCGTCCCTGCATCACCCGTTCTAGGGCCTCTTGCACCAAGGTTTCGGACTCGGAATCAAGCGCCGAAGTCGCCTCATCCAGAATTAAAATGCGTGGATCCTCTACGCCGGACGCATCGTGGCCGGCATCACCGGCGCCACAGGTGCGGTTGCTGGCGCCTATATCGCCGACATCACCGATGGGGAAGATCGGGCTCGCCACTTCGGGCTCATGAGCGCTTGTTTCGGCGTGGGTATGGTGGCAGGCCCCGTGGCCGGGGGACTGTTGGGCGCCATCTCCTTGCATGCACCATTCCTTGCGGCGGCGGTGCTCAACGGCCTCAACCTACTACTGGGCTGCTTCCTAATGCAGGAGTCGCATAAGGGAGAGCGTCGATCGACCGATGCCCTTGAGAGCCTTCAACCCAGTCAGCTCCTTCCGGTGGGCGCGGGGCATGACTATCGTCGCCGCACTTATGACTGTCTTCTTTATCATGCAACTCGTAGGACAGGTGCCGGCAGCGCTCTGGGTCATTTTCGGCGAGGACCGCTTTCGCTGGAGCGCGACGATGATCGGCCTGTCGCTTGCGGTATTCGGAATCTTGCACGCCCTCGCTCAAGCCTTCGTCACTGGTCCCGCCACCAAACGTTTCGGCGAGAAGCAGGCCATTATCGCCGGCATGGCGGCCGACGCGCTGGGCTACGTCTTGCTGGCGTTCGCGACGCGAGGCTGGATGGCCTTCCCCATTATGATTCTTCTCGCTTCCGGCGGCATCGGGATGCCCGCGTTGCAGGCCATGCTGTCCAGGCAGGTAGATGACGACCATCAGGGACAGCTTCAAGGATCGCTCGCGGCTCTTACCAGCCTAACTTCGATCACTGGACCGCTGATCGTCACGGCGATTTATGCCGCCTCGGCGAGCACATGGAACGGGTTGGCATGGATTGTAGGCGCCGCCCTATACCTTGTCTGCCTCCCCGCGTTGCGTCGCGGTGCATGGAGCCGGGCCACCTCGACCTGAATGGAAGCCGGCGGCACCTCGCTAACGGATTCACCACTCCAAGAATTGGAGCCAATCAATTCTTGCGGAGAACTGTGAATGCGCAAACCAACCCTTGGCAGAACATATCCATCGCGTCCGCCATCTCCAGCAGCCGCACGCGGCGCATCTCGGGCAGCGTTGGGTCCTGGCCACGGGTGCGCATGATCGTGCTCCTGTCGTTGAGGACCCGGCTAGGCTGGCGGGGTTGCCTTACTGGTTAGCAGAATGAATCACCGATACGCGAGCGAACGTGAAGCGACTGCTGCTGCAAAACGTCTGCGACCTGAGCAACAACATGAATGGTCTTCGGTTTCCGTGTTTCGTAAAGTCTGGAAACGCGGAAGTCAGCGCCCTGCACCATTATGTTCCGGATCTGCATCGCAGGATGCTGCTGGCTACCCTGTGGAACACCTACATCTGTATTAACGAAGCGCTGGCATTGACCCTGAGTGATTTTTCTCTGGTCCCGCCGCATCCATACCGCCAGTTGTTTACCCTCACAACGTTCCAGTAACCGGGCATGTTCATCATCAGTAACCCGTATCGTGAGCATCCTCTCTCGTTTCATCGGTATCATTACCCCCATGAACAGAAATCCCCCTTACACGGAGGCATCAGTGACCAAACAGGAAAAAACCGCCCTTAACATGGCCCGCTTTATCAGAAGCCAGACATTAACGCTTCTGGAGAAACTCAACGAGCTGGACGCGGATGAACAGGCAGACATCTGTGAATCGCTTCACGACCACGCTGATGAGCTTTACCGCAGCTGCCTCGCGCGTTTCGGTGATGACGGTGAAAACCTCTGACACATGCAGCTCCCGGAGACGGTCACAGCTTGTCTGTAAGCGGATGCCGGGAGCAGACAAGCCCGTCAGGGCGCGTCAGCGGGTGTTGGCGGGTGTCGGGGCGCAGCCATGACCCAGTCACGTAGCGATAGCGGAGTGTATACTGGCTTAACTATGCGGCATCAGAGCAGATTGTACTGAGAGTGCACCATATGCGGTGTGAAATACCGCACAGATGCGTAAGGAGAAAATACCGCATCAGGCGCTCTTCCGCTTCCTCGCTCACTGACTCGCTGCGCTCGGTCGTTCGGCTGCGGCGAGCGGTATCAGCTCACTCAAAGGCGGTAATACGGTTATCCACAGAATCAGGGGATAACGCAGGAAAGAACATGTGAGCAAAAGGCCAGCAAAAGGCCAGGAACCGTAAAAAGGCCGCGTTGCTGGCGTTTTTCCATAGGCTCCGCCCCCCTGACGAGCATCACAAAAATCGACGCTCAAGTCAGAGGTGGCGAAACCCGACAGGACTATAAAGATACCAGGCGTTTCCCCCTGGAAGCTCCCTCGTGCGCTCTCCTGTTCCGACCCTGCCGCTTACCGGATACCTGTCCGCCTTTCTCCCTTCGGGAAGCGTGGCGCTTTCTCATAGCTCACGCTGTAGGTATCTCAGTTCGGTGTAGGTCGTTCGCTCCAAGCTGGGCTGTGTGCACGAACCCCCCGTTCAGCCCGACCGCTGCGCCTTATCCGGTAACTATCGTCTTGAGTCCAACCCGGTAAGACACGACTTATCGCCACTGGCAGCAGCCACTGGTAACAGGATTAGCAGAGCGAGGTATGTAGGCGGTGCTACAGAGTTCTTGAAGTGGTGGCCTAACTACGGCTACACTAGAAGGACAGTATTTGGTATCTGCGCTCTGCTGAAGCCAGTTACCTTCGGAAAAAGAGTTGGTAGCTCTTGATCCGGCAAACAAACCACCGCTGGTAGCGGTGGTTTTTTTGTTTGCAAGCAGCAGATTACGCGCAGAAAAAAAGGATCTCAAGAAGATCCTTTGATCTTTTCTACGGGGTCTGACGCTCAGTGGAACGAAAACTCACGTTAAGGGATTTTGGTCATGAGATTATCAAAAAGGATCTTCACCTAGATCCTTTTAAATTAAAAATGAAGTTTTAAATCAATCTAAAGTATATATGAGTAAACTTGGTCTGACAGTTACCAATGCTTAATCAGTGAGGCACCTATCTCAGCGATCTGTCTATTTCGTTCATCCATAGTTGCCTGACTCCCCGTCGTGTAGATAACTACGATACGGGAGGGCTTACCATCTGGCCCCAGTGCTGCAATGATACCGCGAGACCCACGCTCACCGGCTCCAGATTTATCAGCAATAAACCAGCCAGCCGGAAGGGCCGAGCGCAGAAGTGGTCCTGCAACTTTATCCGCCTCCATCCAGTCTATTAATTGTTGCCGGGAAGCTAGAGTAAGTAGTTCGCCAGTTAATAGTTTGCGCAACGTTGTTGCCATTGCTGCAGGCATCGTGGTGTCACGCTCGTCGTTTGGTATGGCTTCATTCAGCTCCGGTTCCCAACGATCAAGGCGAGTTACATGATCCCCCATGTTGTGCAAAAAAGCGGTTAGCTCCTTCGGTCCTCCGATCGTTGTCAGAAGTAAGTTGGCCGCAGTGTTATCACTCATGGTTATGGCAGCACTGCATAATTCTCTTACTGTCATGCCATCCGTAAGATGCTTTTCTGTGACTGGTGAGTACTCAACCAAGTCATTCTGAGAATAGTGTATGCGGCGACCGAGTTGCTCTTGCCCGGCGTCAACACGGGATAATACCGCGCCACATAGCAGAACTTTAAAAGTGCTCATCATTGGAAAACGTTCTTCGGGGCGAAAACTCTCAAGGATCTTACCGCTGTTGAGATCCAGTTCGATGTAACCCACTCGTGCACCCAACTGATCTTCAGCATCTTTTACTTTCACCAGCGTTTCTGGGTGAGCAAAAACAGGAAGGCAAAATGCCGCAAAAAAGGGAATAAGGGCGACACGGAAATGTTGAATACTCATACTCTTCCTTTTTCAATATTATTGAAGCATTTATCAGGGTTATTGTCTCATGAGCGGATACATATTTGAATGTATTTAGAAAAATAAACAAATAGGGGTTCCGCGCACATTTCCCCGAAAAGTGCCACCTGACGTCTAAGAAACCATTATTATCATGACATTAACCTATAAAAATAGGCGTATCACGAGGCCCTTTCGTCTTCAAGAATT

## **File S4:** Plasmid map and sequence of pWX1221


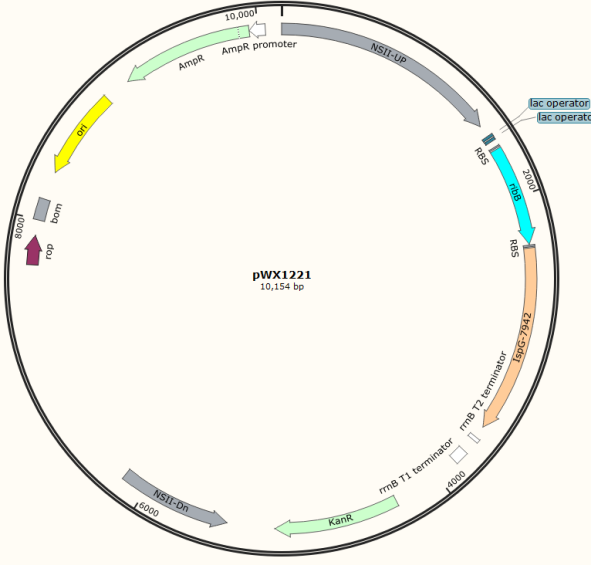


## Sequence:

AGCTTGTCATCTGCCGGATGAGGCAAAACCCTGCCTACGGCGCGATTACATCGTCCCAGCGCGATCGCTCTTACTGTTGATGGCTCGTGCTTAAAAACAATGCAAACTTCACCGTTTCAGCTGGTGATTTTCGACTGTGATGGTGTGCTTGTTGATAGCGAACGCATCACTAATCGCGTCTTTGCAGACATGCTCAATGAACTGGGTCTGTTGGTGACTTTGGATGACATGTTTGAGCAGTTTGTGGGTCATTCCATGGCTGACTGTCTCAAACTAATTGAGCGACGGTTAGGCAATCCTCCACCCCCTGACTTTGTTCAGCACTATCAACGCCGTACCCGTATCGCGTTAGAAACGCATCTACAAGCCGTTCCTGGGGTTGAAGAGGCTTTGGATGCTCTTGAATTGCCCTACTGTGTTGCGTCCAGTGGTGATCATCAAAAGATGCGAACCACACTGAGCCTGACGAAGCTCTGGCCACGATTTGAGGGACGAATCTTCAGCGTGACTGAAGTACCTCGCGGCAAGCCATTTCCCGATGTCTTTTTGTTGGCCGCCGATCGCTTCGGGGTTAATCCTACGGCCTGCGCTGTGATCGAAGACACCCCCTTGGGAGTAGCGGCAGGCGTGGCGGCAGGAATGCAAGTGTTTGGCTACGCGGGTTCCATGCCCGCTTGGCGTCTGCAAGAAGCCGGTGCCCATCTCATTTTTGACGATATGCGACTGCTGCCCAGTCTGCTCCAATCGTCGCCAAAAGATAACTCCACAGCATTGCCCAATCCCTAACCCCTGCTCGCGCCGCAACTACACACTAAACCGTTCCTGCGCGATCGCTCTTACTGTTGATGGCTCGTGCTTAAAAACAATGCAACCCTAACCGTTTCAGCTGGTGATTTTCGGACGATTTGGCTTACAGGGATAACTGAGAGTCAACAGCCTCTGTCCGTCATTGCACACCCATCCATGCACTGGGGACTTGACTCATGCTGAATCACATTTCCCTTGTCCATTGGGCGAGAGGGGAGGGGAATCTTCTGGACTCTTCACTAAGCGGCGATCGCAGGTTCTTCTACCCAAGCAGTGGCGATCGCTTGATTGCAGTCTTCAATGCTGGCCTCTGCAGCCATCGCCGCCACCAAAGCATCGTAGGCGGGACGTTGTTGCTCCAGTAAAGTCTTCGCCCGTAACAATCCCCAGCGACTGCGTAAATCCGCTTCGGCAGGATTGCGATCGAGTTGCCGCCACAGTTGTTTCCACTGGGCGCGATCGTCAGCTCCCCCTTCCACGTTGCCGTAGACCAGTTGCTCTGCCGCTGCACCGGCCATCAACACCTGACACCACTGTTCCAGCGATCGCTGACTGAGTTGCCCCTGTGCGGCTTCGGCTTCTAGCGCAGCTGCTTGGAACTGCACACCCCCGCGACCAGGTTGTCCTTGGCGCAGCGCTTCCCACGCTGAGAGGGTGTAGCCCGTCACGGGTAACCGATTAAGAAACCATTATTATCATGACATTAACCTATAAAAATAGGCGTATCACGAGGCCCTTTCGTCTTCACCTCGAGAATTGTGAGCGGATAACAATTGACATTGTGAGCGGATAACAAGATACTGAGCACATCAGCAGGACGCACTGACCGAATTCATTAAAGAGGAGAAAGATATCATGAACCAGACTTTGCTCAGCAGTTTTGGGACACCATTCGAACGAGTGGAAAATGCGCTGGCGGCATTGCGGGAAGGACGGGGCGTCATGGTGCTCGATGATGAGGATCGCGAAAATGAAGGTGACATGATCTTTCCTGCCGAAACCATGACCGTGGAGCAAATGGCGTTGACCATTCGCCATGGGTCCGGTATTGTGTGTTTGTGCATCACTGAGGATCGCCGGAAGCAGCTGGATTTGCCGATGATGGTCGAAAACAACACGTCGGCCTACGGCACGGGTTTTACAGTCACGATTGAAGCTGCGGAAGGTGTGACCACAAGCGTCTCTGCGGCTGATCGTATTACAACCGTGCGCGCCGCCATCGCAGATGGTGCGAAGCCCTCCGATCTGAATCGACCTGGTCACGTGTTCCCTCTGCGTGCCCAAGCGGGCGGGGTGCTGACACGCGGCGGGCACACTGAAGCCACAATCGACCTGATGACGCTCGCCGGATTTAAACCCGCGGGAGTTCTGTGCGAGCTGACGAATGACGATGGTACTATGGCCCGCGCCCCCGAGTGTATTGAGTTCGCTAACAAACACAATATGGCACTGGTGACAATTGAAGATCTGGTCGCGTACCGCCAAGCACACGAGCGAAAAGCATCTTAAGTCGACAAAGAGGAGAAAgatatcATGCAGACCCTCTCCACCCCCAGCACAACTGCCACTGAGTTTGATACCGTCATCCACCGGCGCCCTACGCGATCGGTTCGGGTCGGTGATATCTGGATTGGCAGCCGCCATCCCGTCGTTGTCCAGTCGATGATCAACGAGGACACCCTCGACATCGATGGGTCTGTTGCCGCGATCCGCCGCCTGCATGAGATTGGCTGCGAGATCGTCCGCGTCACGGTGCCCAGTCTTGGCCATGCCAAAGCGGTCGGTGACATTAAAAAGAAACTGCAAGACACCTATCGCGACGTGCCCTTGGTTGCCGACGTGCACCACAACGGTATGAAGATCGCGCTGGAAGTCGCCAAGCACGTTGACAAAGTGCGGATCAATCCTGGTCTCTACGTCTTTGAGAAGCCCGATCCGAATCGTCAGGGCTACACACCAGAAGAATTTGAGCGAATTGGCAAGCAAATTCGCGACACGCTTGAGCCCTTAGTCACCAGCCTGCGCGAGCAGGACAAGGCCATGCGGATTGGCGTCAACCACGGCTCTCTAGCCGAGCGGATGCTGTTCACCTACGGCGACACGCCGGAAGGCATGGTGGAATCGGCGTTGGAATTCCTGCGACTCTGCGAAGAGATGGACTTCCGTAATCTCGTCATCTCCATGAAAGCGAGCCGAGCGCCGGTGATGATGGCGGCCTATCGCCTGATGGCCAAACGGATGGATGATCTGGGCATGGATTATCCGCTCCACTTGGGTGTGACCGAAGCCGGTGATGGTGACTATGGCCGGATCAAATCGACGGTTGGGATTGGCACGCTGCTAGCGGAAGGGATTGGCGATACGATTCGCGTTTCGCTCACGGAAGCCCCCGAAAACGAAATTCCGGTTTGCTATTCGATTTTGCAAGCCCTCGGCCTCCGCAAAACCATGGTTGAGTATGTCGCCTGTCCAAGTTGCGGACGGACACTCTTCAATCTGGAAGAGGTCCTTCACAAAGTGCGGGCTGCAACCAATCATTTGGTTGGCTTGGATATTGCTGTCATGGGTTGCATCGTCAATGGCCCTGGCGAAATGGCTGATGCTGACTACGGCTACGTTGGCAAAACCCCAGGAACGATCGCCCTTTACCGAGGACGAGATGAAATCAAACGAGTTCCCGAAGAGCAAGGTGTAGAAGAACTGATCAACCTGATCAAAGCAGATGGACGCTGGGTTGAACCGGAACCGATTGCCTAAGTCGACAGGCCTCTAGACCCGGGCTCGAGCTAGCAAGCTTGGCCGGATCCGGCCGGATCCGGAGTTTGTAGAAACGCAAAAAGGCCATCCGTCAGGATGGCCTTCTGCTTAATTTGATGCCTGGCAGTTTATGGCGGGCGTCCTGCCCGCCACCCTCCGGGCCGTTGCTTCGCAACGTTCAAATCCGCTCCCGGCGGATTTGTCCTACTCAGGAGAGCGTTCACCGACAAACAACAGATAAAACGAAAGGCCCAGTCTTTCGACTGAGCCTTTCGTTTTATTTGATGCCTGGCAGTTCCCTACTCTCGCATGGGGAGACCCCACACTACCATCGGCGCTACGGCGTTTCACTTCTGAGTTCGGCATGGGGTCAGGTGGGACCACCGCGCTACTGCCGCCAGGCAAATTCTGTTTTATCAGCCGTTACCCCACCTACTAGCTAATCCCATCTGGGCACATCCGATGGCAAGAGGCCCGAAGGTCCCCCTCTTTGGTCTTGCGACGTTATGCGGTATTAGCTACCGTTTCCAGTAGTTATCCCCCTCCATCAGGCAGTTTCCCAGACATTACTCACCCGTCCGCCACTCGTCAGCAAAGAAGCAAGCTTAGATCGACCTGCAGGGGGGGGGGGGAAAGCCACGTTGTGTCTCAAAATCTCTGATGTTACATTGCACAAGATAAAAATATATCATCATGAACAATAAAACTGTCTGCTTACATAAACAGTAATACAAGGGGTGTTATGAGCCATATTCAACGGGAAACGTCTTGCTCGAGGCCGCGATTAAATTCCAACATGGATGCTGATTTATATGGGTATAAATGGGCTCGCGATAATGTCGGGCAATCAGGTGCGACAATCTATCGATTGTATGGGAAGCCCGATGCGCCAGAGTTGTTTCTGAAACATGGCAAAGGTAGCGTTGCCAATGATGTTACAGATGAGATGGTCAGACTAAACTGGCTGACGGAATTTATGCCTCTTCCGACCATCAAGCATTTTATCCGTACTCCTGATGATGCATGGTTACTCACCACTGCGATCCCCGGGAAAACAGCATTCCAGGTATTAGAAGAATATCCTGATTCAGGTGAAAATATTGTTGATGCGCTGGCAGTGTTCCTGCGCCGGTTGCATTCGATTCCTGTTTGTAATTGTCCTTTTAACAGCGATCGCGTATTTCGTCTCGCTCAGGCGCAATCACGAATGAATAACGGTTTGGTTGATGCGAGTGATTTTGATGACGAGCGTAATGGCTGGCCTGTTGAACAAGTCTGGAAAGAAATGCATAAGCTTTTGCCATTCTCACCGGATTCAGTCGTCACTCATGGTGATTTCTCACTTGATAACCTTATTTTTGACGAGGGGAAATTAATAGGTTGTATTGATGTTGGACGAGTCGGAATCGCAGACCGATACCAGGATCTTGCCATCCTATGGAACTGCCTCGGTGAGTTTTCTCCTTCATTACAGAAACGGCTTTTTCAAAAATATGGTATTGATAATCCTGATATGAATAAATTGCAGTTTCATTTGATGCTCGATGAGTTTTTCTAATCAGAATTGGTTAATTGGTTGTAACACTGGCAGAGCATTACGCTGACTTGACGGGACGGCGGCTTTGTTGAATAAATCGAACTTTTGCTGAGTTGAAGGATCAGATCACGCATCTTCCCGACAACGCAGACCGTTCCGTGGCAAAGCAAAAGTTCAAAATCACCAACTGGTCCACCTACAACAAAGCTCTCATCAACCGTGGCTCCCTCACTTTCTGGCTGGATGATGGGGCGATTCAGGCCTGGTATGAGTCAGCAACACCTTCTTCACGAGGCAGACCTCAGCGCCCCCCCCCCCCTGCAGGTCGATCTGGTAACCCCAGCGCGGTTGCTACCAAGTAGTGACCCGCTTCGTGATGCAAAATCCGCTGACGATATTCGGGCGATCGCTGCTGAATGCCATCGAGCAGTAACGTGGCACCCCGCCCCTGCCAAGTCACCGCATCCAGACTGAACAGCACCAAGAGGCTAAAACCCAATCCCGCCGGTAGCAGCGGAGAACTACCCAGCATTGGTCCCACCAAAGCTAATGCCGTCGTGGTAAAAATCGCGATCGCCGTCAGACTCAAGCCCAGTTCGCTCATGCTTCCTCATCTAGGTCACAGTCTTCGGCGATCGCATCGATCTGATGCTGCAGCAAGCGTTTTCCATACCGGCGATCGCGCCGTCGCCCTTTCGCTGCCGTGGCCCGCTTACGAGCTCGTTTATCGACCACGATCGCATCCAAATCCGCGATCGCTTCCCAGTCCGGCAATTCAGTCTGGGGCGTCCGTTTCATTAATCCTGATCAGGCACGAAATTGCTGTGCGTAGTATCGCGCATAGCGGCCAGCCTCTGCCAACAGCGCATCGTGATTGCCTGCCTCAACAATCTGGCCGCGCTCCATCACCAAGATGCGGCTGGCATTACGAACCGTAGCCAGACGGTGAGCAATGATAAAGACCGTCCGTCCCTGCATCACCCGTTCTAGGGCCTCTTGCACCAAGGTTTCGGACTCGGAATCAAGCGCCGAAGTCGCCTCATCCAGAATTAAAATGCGTGGATCCTCTACGCCGGACGCATCGTGGCCGGCATCACCGGCGCCACAGGTGCGGTTGCTGGCGCCTATATCGCCGACATCACCGATGGGGAAGATCGGGCTCGCCACTTCGGGCTCATGAGCGCTTGTTTCGGCGTGGGTATGGTGGCAGGCCCCGTGGCCGGGGGACTGTTGGGCGCCATCTCCTTGCATGCACCATTCCTTGCGGCGGCGGTGCTCAACGGCCTCAACCTACTACTGGGCTGCTTCCTAATGCAGGAGTCGCATAAGGGAGAGCGTCGATCGACCGATGCCCTTGAGAGCCTTCAACCCAGTCAGCTCCTTCCGGTGGGCGCGGGGCATGACTATCGTCGCCGCACTTATGACTGTCTTCTTTATCATGCAACTCGTAGGACAGGTGCCGGCAGCGCTCTGGGTCATTTTCGGCGAGGACCGCTTTCGCTGGAGCGCGACGATGATCGGCCTGTCGCTTGCGGTATTCGGAATCTTGCACGCCCTCGCTCAAGCCTTCGTCACTGGTCCCGCCACCAAACGTTTCGGCGAGAAGCAGGCCATTATCGCCGGCATGGCGGCCGACGCGCTGGGCTACGTCTTGCTGGCGTTCGCGACGCGAGGCTGGATGGCCTTCCCCATTATGATTCTTCTCGCTTCCGGCGGCATCGGGATGCCCGCGTTGCAGGCCATGCTGTCCAGGCAGGTAGATGACGACCATCAGGGACAGCTTCAAGGATCGCTCGCGGCTCTTACCAGCCTAACTTCGATCACTGGACCGCTGATCGTCACGGCGATTTATGCCGCCTCGGCGAGCACATGGAACGGGTTGGCATGGATTGTAGGCGCCGCCCTATACCTTGTCTGCCTCCCCGCGTTGCGTCGCGGTGCATGGAGCCGGGCCACCTCGACCTGAATGGAAGCCGGCGGCACCTCGCTAACGGATTCACCACTCCAAGAATTGGAGCCAATCAATTCTTGCGGAGAACTGTGAATGCGCAAACCAACCCTTGGCAGAACATATCCATCGCGTCCGCCATCTCCAGCAGCCGCACGCGGCGCATCTCGGGCAGCGTTGGGTCCTGGCCACGGGTGCGCATGATCGTGCTCCTGTCGTTGAGGACCCGGCTAGGCTGGCGGGGTTGCCTTACTGGTTAGCAGAATGAATCACCGATACGCGAGCGAACGTGAAGCGACTGCTGCTGCAAAACGTCTGCGACCTGAGCAACAACATGAATGGTCTTCGGTTTCCGTGTTTCGTAAAGTCTGGAAACGCGGAAGTCAGCGCCCTGCACCATTATGTTCCGGATCTGCATCGCAGGATGCTGCTGGCTACCCTGTGGAACACCTACATCTGTATTAACGAAGCGCTGGCATTGACCCTGAGTGATTTTTCTCTGGTCCCGCCGCATCCATACCGCCAGTTGTTTACCCTCACAACGTTCCAGTAACCGGGCATGTTCATCATCAGTAACCCGTATCGTGAGCATCCTCTCTCGTTTCATCGGTATCATTACCCCCATGAACAGAAATCCCCCTTACACGGAGGCATCAGTGACCAAACAGGAAAAAACCGCCCTTAACATGGCCCGCTTTATCAGAAGCCAGACATTAACGCTTCTGGAGAAACTCAACGAGCTGGACGCGGATGAACAGGCAGACATCTGTGAATCGCTTCACGACCACGCTGATGAGCTTTACCGCAGCTGCCTCGCGCGTTTCGGTGATGACGGTGAAAACCTCTGACACATGCAGCTCCCGGAGACGGTCACAGCTTGTCTGTAAGCGGATGCCGGGAGCAGACAAGCCCGTCAGGGCGCGTCAGCGGGTGTTGGCGGGTGTCGGGGCGCAGCCATGACCCAGTCACGTAGCGATAGCGGAGTGTATACTGGCTTAACTATGCGGCATCAGAGCAGATTGTACTGAGAGTGCACCATATGCGGTGTGAAATACCGCACAGATGCGTAAGGAGAAAATACCGCATCAGGCGCTCTTCCGCTTCCTCGCTCACTGACTCGCTGCGCTCGGTCGTTCGGCTGCGGCGAGCGGTATCAGCTCACTCAAAGGCGGTAATACGGTTATCCACAGAATCAGGGGATAACGCAGGAAAGAACATGTGAGCAAAAGGCCAGCAAAAGGCCAGGAACCGTAAAAAGGCCGCGTTGCTGGCGTTTTTCCATAGGCTCCGCCCCCCTGACGAGCATCACAAAAATCGACGCTCAAGTCAGAGGTGGCGAAACCCGACAGGACTATAAAGATACCAGGCGTTTCCCCCTGGAAGCTCCCTCGTGCGCTCTCCTGTTCCGACCCTGCCGCTTACCGGATACCTGTCCGCCTTTCTCCCTTCGGGAAGCGTGGCGCTTTCTCATAGCTCACGCTGTAGGTATCTCAGTTCGGTGTAGGTCGTTCGCTCCAAGCTGGGCTGTGTGCACGAACCCCCCGTTCAGCCCGACCGCTGCGCCTTATCCGGTAACTATCGTCTTGAGTCCAACCCGGTAAGACACGACTTATCGCCACTGGCAGCAGCCACTGGTAACAGGATTAGCAGAGCGAGGTATGTAGGCGGTGCTACAGAGTTCTTGAAGTGGTGGC

CTAACTACGGCTACACTAGAAGGACAGTATTTGGTATCTGCGCTCTGCTGAAGCCAGTTACCTTCGGAAAAAGAGTTGGTAGCTCTTGATCCGGCAAACAAACCACCGCTGGTAGCGGTGGTTTTTTTGTTTGCAAGCAGCAGATTACGCGCAGAAAAAAAGGATCTCAAGAAGATCCTTTGATCTTTTCTACGGGGTCTGACGCTCAGTGGAACGAAAACTCACGTTAAGGGATTTTGGTCATGAGATTATCAAAAAGGATCTTCACCTAGATCCTTTTAAATTAAAAATGAAGTTTTAAATCAATCTAAAGTATATATGAGTAAACTTGGTCTGACAGTTACCAATGCTTAATCAGTGAGGCACCTATCTCAGCGATCTGTCTATTTCGTTCATCCATAGTTGCCTGACTCCCCGTCGTGTAGATAACTACGATACGGGAGGGCTTACCATCTGGCCCCAGTGCTGCAATGATACCGCGAGACCCACGCTCACCGGCTCCAGATTTATCAGCAATAAACCAGCCAGCCGGAAGGGCCGAGCGCAGAAGTGGTCCTGCAACTTTATCCGCCTCCATCCAGTCTATTAATTGTTGCCGGGAAGCTAGAGTAAGTAGTTCGCCAGTTAATAGTTTGCGCAACGTTGTTGCCATTGCTGCAGGCATCGTGGTGTCACGCTCGTCGTTTGGTATGGCTTCATTCAGCTCCGGTTCCCAACGATCAAGGCGAGTTACATGATCCCCCATGTTGTGCAAAAAAGCGGTTAGCTCCTTCGGTCCTCCGATCGTTGTCAGAAGTAAGTTGGCCGCAGTGTTATCACTCATGGTTATGGCAGCACTGCATAATTCTCTTACTGTCATGCCATCCGTAAGATGCTTTTCTGTGACTGGTGAGTACTCAACCAAGTCATTCTGAGAATAGTGTATGCGGCGACCGAGTTGCTCTTGCCCGGCGTCAACACGGGATAATACCGCGCCACATAGCAGAACTTTAAAAGTGCTCATCATTGGAAAACGTTCTTCGGGGCGAAAACTCTCAAGGATCTTACCGCTGTTGAGATCCAGTTCGATGTAACCCACTCGTGCACCCAACTGATCTTCAGCATCTTTTACTTTCACCAGCGTTTCTGGGTGAGCAAAAACAGGAAGGCAAAATGCCGCAAAAAAGGGAATAAGGGCGACACGGAAATGTTGAATACTCATACTCTTCCTTTTTCAATATTATTGAAGCATTTATCAGGGTTATTGTCTCATGAGCGGATACATATTTGAATGTATTTAGAAAAATAAACAAATAGGGGTTCCGCGCACATTTCCCCGAAAAGTGCCACCTGACGTCTAAGAAACCATTATTATCATGACATTAACCTATAAAAATAGGCGTATCACGAGGCCCTTTCGTCTTCAAGAATT

## **File S5:** Plasmid map and sequence of pLM2211


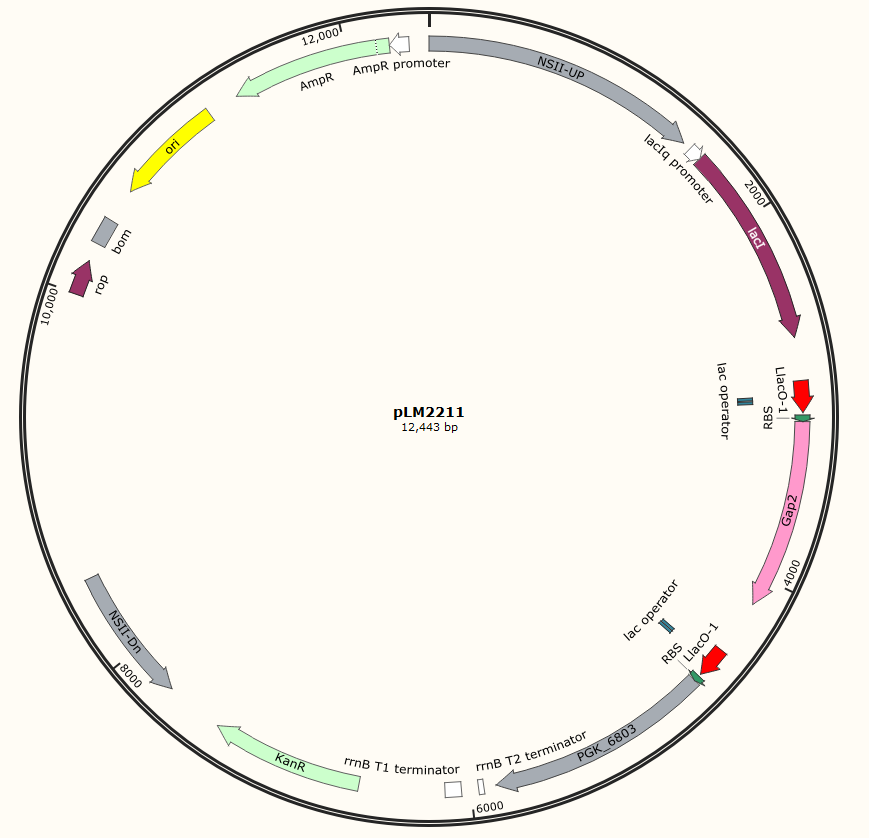


## Sequence:

AGCTTGTCATCTGCCGGATGAGGCAAAACCCTGCCTACGGCGCGATTACATCGTCCCAGCGCGATCGCTCTTACTGTTGATGGCTCGTGCTTAAAAACAATGCAAACTTCACCGTTTCAGCTGGTGATTTTCGACTGTGATGGTGTGCTTGTTGATAGCGAACGCATCACTAATCGCGTCTTTGCAGACATGCTCAATGAACTGGGTCTGTTGGTGACTTTGGATGACATGTTTGAGCAGTTTGTGGGTCATTCCATGGCTGACTGTCTCAAACTAATTGAGCGACGGTTAGGCAATCCTCCACCCCCTGACTTTGTTCAGCACTATCAACGCCGTACCCGTATCGCGTTAGAAACGCATCTACAAGCCGTTCCTGGGGTTGAAGAGGCTTTGGATGCTCTTGAATTGCCCTACTGTGTTGCGTCCAGTGGTGATCATCAAAAGATGCGAACCACACTGAGCCTGACGAAGCTCTGGCCACGATTTGAGGGACGAATCTTCAGCGTGACTGAAGTACCTCGCGGCAAGCCATTTCCCGATGTCTTTTTGTTGGCCGCCGATCGCTTCGGGGTTAATCCTACGGCCTGCGCTGTGATCGAAGACACCCCCTTGGGAGTAGCGGCAGGCGTGGCGGCAGGAATGCAAGTGTTTGGCTACGCGGGTTCCATGCCCGCTTGGCGTCTGCAAGAAGCCGGTGCCCATCTCATTTTTGACGATATGCGACTGCTGCCCAGTCTGCTCCAATCGTCGCCAAAAGATAACTCCACAGCATTGCCCAATCCCTAACCCCTGCTCGCGCCGCAACTACACACTAAACCGTTCCTGCGCGATCGCTCTTACTGTTGATGGCTCGTGCTTAAAAACAATGCAACCCTAACCGTTTCAGCTGGTGATTTTCGGACGATTTGGCTTACAGGGATAACTGAGAGTCAACAGCCTCTGTCCGTCATTGCACACCCATCCATGCACTGGGGACTTGACTCATGCTGAATCACATTTCCCTTGTCCATTGGGCGAGAGGGGAGGGGAATCTTCTGGACTCTTCACTAAGCGGCGATCGCAGGTTCTTCTACCCAAGCAGTGGCGATCGCTTGATTGCAGTCTTCAATGCTGGCCTCTGCAGCCATCGCCGCCACCAAAGCATCGTAGGCGGGACGTTGTTGCTCCAGTAAAGTCTTCGCCCGTAACAATCCCCAGCGACTGCGTAAATCCGCTTCGGCAGGATTGCGATCGAGTTGCCGCCACAGTTGTTTCCACTGGGCGCGATCGTCAGCTCCCCCTTCCACGTTGCCGTAGACCAGTTGCTCTGCCGCTGCACCGGCCATCAACACCTGACACCACTGTTCCAGCGATCGCTGACTGAGTTGCCCCTGTGCGGCTTCGGCTTCTAGCGCAGCTGCTTGGAACTGCACACCCCCGCGACCAGGTTGTCCTTGGCGCAGCGCTTCCCACGCTGAGAGGGTGTAGCCCGTCACGGGTAACCAGATCAATTCGCGCGCGAAGGCGAAGCGGCATGCATTTACGTTGACACCATCGAATGGTGCAAAACCTTTCGCGGTATGGCATGATAGCGCCCGGAAGAGAGTCAATTCAGGGTGGTGAATGTGAAACCAGTAACGTTATACGATGTCGCAGAGTATGCCGGTGTCTCTTATCAGACCGTTTCCCGCGTGGTGAACCAGGCCAGCCACGTTTCTGCGAAAACGCGGGAAAAAGTGGAAGCGGCGATGGCGGAGCTGAATTACATTCCCAACCGCGTGGCACAACAACTGGCGGGCAAACAGTCGTTGCTGATTGGCGTTGCCACCTCCAGTCTGGCCCTGCACGCGCCGTCGCAAATTGTCGCGGCGATTAAATCTCGCGCCGATCAACTGGGTGCCAGCGTGGTGGTGTCGATGGTAGAACGAAGCGGCGTCGAAGCCTGTAAAGCGGCGGTGCACAATCTTCTCGCGCAACGCGTCAGTGGGCTGATCATTAACTATCCGCTGGATGACCAGGATGCCATTGCTGTGGAAGCTGCCTGCACTAATGTTCCGGCGTTATTTCTTGATGTCTCTGACCAGACACCCATCAACAGTATTATTTTCTCCCATGAAGACGGTACGCGACTGGGCGTGGAGCATCTGGTCGCATTGGGTCACCAGCAAATCGCGCTGTTAGCGGGCCCATTAAGTTCTGTCTCGGCGCGTCTGCGTCTGGCTGGCTGGCATAAATATCTCACTCGCAATCAAATTCAGCCGATAGCGGAACGGGAAGGCGACTGGAGTGCCATGTCCGGTTTTCAACAAACCATGCAAATGCTGAATGAGGGCATCGTTCCCACTGCGATGCTGGTTGCCAACGATCAGATGGCGCTGGGCGCAATGCGCGCCATTACCGAGTCCGGGCTGCGCGTTGGTGCGGATATCTCGGTAGTGGGATACGACGATACCGAAGACAGCTCATGTTATATCCCGCCGTCAACCACCATCAAACAGGATTTTCGCCTGCTGGGGCAAACCAGCGTGGACCGCTTGCTGCAACTCTCTCAGGGCCAGGCGGTGAAGGGCAATCAGCTGTTGCCCGTCTCACTGGTGAAAAGAAAAACCACCCTGGCGCCCAATACGCAAACCGCCTCTCCCCGCGCGTTGGCCGATTCATTAATGCAGCTGGCACGACAGGTTTCCCGACTGGAAAGCGGGCAGTGAGCGCAACGCAATTAATGTGAGTTAGCGCGAATTGATCTGGTTTGACAGCTTATCATCGACTGCACGGTGCACCAATGCTTCTGGCGTCAGGCAGCCATCGGAAGCTGTGGTATGGCTGTGCAGGTCGTAAATCACTGCATAATTCGTGTCGCTCAAGGCGCACTCCCGTTCTGGATAATGTTTTTTGCGCCGACATCATAACGGTTCTGGCAAATATTCTGAAATGAGCTGTAAGAAACCATTATTATCATGACATTAACCTATAAAAATAGGCGTATCACGAGGCCCTTTCGTCTTCACCTCGAGAATTGTGAGCGGATAACAATTGACATTGTGAGCGGATAACAAGATACTGAGCACATCAGCAGGACGCACTGACCGAATTCATTAAAGAGGAGAAAGATATCGTCGACTTTACCGTTCCCAAAAATAAAGAAGGAGGAACAGCATGACTAGAGTAGCAATTAACGGATTTGGACGGATCGGACGCAACTTTCTCCGTTGCTGGCTGGGGCGCACCGATAGCCAGTTAGAAGTAGTCGGTATCAACGACACCTCTGATCCCAGAACCAATGCTCACCTTTTGCGCTACGACTCCATGTTGGGTAAGTTGGATGCGGACATCAGTGCCGACGAAAACTCCATTACCGTCAATGGCAAGACTATTAAATGTGTTTCCGACCGGAATCCCCTCAATTTGCCCTGGGCAGAATGGAATGTAGATCTAGTCATCGAAGCCACCGGTGTTTTTGTTACCCATGAAGGGGCCACCAAGCACGTTCAAGCCGGAGCCAAAAAAGTTTTAATCACTGCTCCTGGCAAGGGGCCCAACATCGGCACCTATGTGGTGGGGGTCAATGCCCACGAATATAAGCACGAAGAATACGAAGTAATTAGTAACGCTAGTTGTACTACCAACTGCCTCGCCCCGATCGCCAAAGTAATCAACGACAATTTTGGCATCATCAAAGGCACCATGACCACCACCCACAGCTACACCGGAGACCAACGGATCCTCGATGCTAGCCACCGGGATCTACGCCGGGCCCGGGCTGCTGCCGTTAACATCGTGCCCACCTCCACCGGAGCTGCCAAAGCGGTGGCCCTGGTAATTCCTGAACTGCAAGGCAAATTGAACGGTATTGCCCTGCGGGTGCCCACCCCCAACGTTTCCGTGGTGGATTTGGTAGTACAAGTAGAGAAAAACACCATCGCTGAACAGGTTAACGGAGTGCTCAAAGAAGCAGCCAACACCAGCCTTAAAGGAGTGTTGGAATACACCGATTTGGAATTGGTTTCCAGCGACTTCCGGGGCACGGATTGCTCTTCCACTGTGGATGGTAGTCTGACCATGGTAATGGGCGGTGACATGGTTAAAGTCATTGCTTGGTACGACAACGAATGGGGCTATTCCCAACGGGTGGTGGACTTGGCTGAAATTGTGGCTAAAAACTGGAAATAGGTCGACAGGCCTCTAGACCCGGGCTCGAGCTAGCAAGCTTGGCCGGATCCGGCCGGATCCGGAGTTTGTAGAAACGCAAAAAGGCCATCCGTCAGGATGGCCTTCTGCTTAATTTGATGCCTGGCAGTTTATGGCGGGCGTCCTGCCCGCCACCCTCCGGGCCGTTGCTTCGCAACGTTCAAATCCGCTCCCGGCGGATTTGTCCTACTCAGGAGAGCGTTCACCGACAAACAACAGATAAAACGAAAGGCCCAGTCTTTCGACTGAGCCTTTCGTTTTATTTGATGCCTGGCTAAGAAACCATTATTATCATGACATTAACCTATAAAAATAGGCGTATCACGAGGCCCTTTCGTCTTCACCTCGAGAATTGTGAGCGGATAACAATTGACATTGTGAGCGGATAACAAGATACTGAGCACATCAGCAGGACGCACTGACCGAATTCATTAAAGAGGAGAAAGATATCGTCGACTTTACCGTTCCCAAAAATAAAGAAGGAGGAACAGCATGTTGTCTAAGCAATCGATCGCCAATTTGACGGAGGCAGACCTCGCAGGGAAACGGGTTTTTGTCCGGGTAGATTTTAATGTGCCCCTAGATAACGGCAGCATCACCGACGACACCAGGATTCGGGCAGCCTTACCCACCATCAAAGACCTGTTGAGCAAAGGCGCCAAAGTTATTTTGGGCAGTCATTTTGGCCGTCCCAAGGGCAAAGTAGTGGACAGCATGCGCCTCACCCCCGTTGGCGATCGCCTAGGGGAATTATTGGGCCAGCCGGTGGTCAAATGCGACGATTGCATTGGTGCTGAAGTTACAGCCAAAATTGCTAGCCTACCCAATGGTGGTGTGGCCCTGTTGGAAAATCTCCGCTTCCATGCCGGGGAAGAAGGTAACGATGCTGAATTTGCCAAAGCTTTAGCGGCCAACGCCGACCTCTACGTTAACGATGCCTTTGGTACTGCCCACCGGGCCCACGCTTCCACCGAAGGGGTCACCCATTTCCTCAGCCCCAACGTTGCTGGTTACCTAATCGAAAAGGAATTACAGTTCCTCCAAGGAGCCATCGAAGCCCCCAAACGTCCCCTAGTGGCGATCGTGGGAGGTTCCAAAGTGTCCAGTAAAATCGGTGTGATCGAAACCCTATTGGACAAGTGCGATAAGTTGATCATCGGCGGCGGCATGATTTTCACCTTCTACAAAGCCCAAGGTTTAAACACCGGCAAATCCCTGGTGGAAGAAGACAAATTGGACTTGGCCAAATCCCTCATGGCTAAAGCCAAAGAAAAAGGCGTGGAATTTCTCCTGCCCACGGACGTAGTAGTGGCCGACAACTTTGCCCCCGATGCCAATGCCCAAACCGTTGGTGTCGATGCAATTCCCGATGGTTGGATGGGTCTAGACATTGGTCCCGACTCCGTCAAAACCTTCCAGGATGCCCTCGCTGGTTGTGGCACTGTCATCTGGAACGGCCCCATGGGGGTATTTGAATTTGACAAATTTGCCGTTGGTACCGAGGCGATCGCCTGCAGCTTGGCTGAATTGACCGCCAGTGGCACTGTCACCATCATCGGTGGTGGAGATTCTGTCGCCGCAGTGGAAAAAGTGGGAGTGGCCGAAAAAATGAGCCATATTTCCACCGGTGGGGGCGCTAGCCTGGAATTGCTAGAAGGTAAAGTTCTGCCCGGCATTGCCGCTTTAGATGACCGATAACTAGACCCGGGCTCGAGCTAGCAAGCTTGGCCGGATCCGGCCGGATCCGGAGTTTGTAGAAACGCAAAAAGGCCATCCGTCAGGATGGCCTTCTGCTTAATTTGATGCCTGGCAGTTTATGGCGGGCGTCCTGCCCGCCACCCTCCGGGCCGTTGCTTCGCAACGTTCAAATCCGCTCCCGGCGGATTTGTCCTACTCAGGAGAGCGTTCACCGACAAACAACAGATAAAACGAAAGGCCCAGTCTTTCGACTGAGCCTTTCGTTTTATTTGATGCCTGGCAGTTCCCTACTCTCGCATGGGGAGACCCCACACTACCATCGGCGCTACGGCGTTTCACTTCTGAGTTCGGCATGGGGTCAGGTGGGACCACCGCGCTACTGCCGCCAGGCAAATTCTGTTTTATCAGCCGTTACCCCACCTACTAGCTAATCCCATCTGGGCACATCCGATGGCAAGAGGCCCGAAGGTCCCCCTCTTTGGTCTTGCGACGTTATGCGGTATTAGCTACCGTTTCCAGTAGTTATCCCCCTCCATCAGGCAGTTTCCCAGACATTACTCACCCGTCCGCCACTCGTCAGCAAAGAAGCAAGCTTAGATCGACCTGCAGGGGGGGGGGGGAAAGCCACGTTGTGTCTCAAAATCTCTGATGTTACATTGCACAAGATAAAAATATATCATCATGAACAATAAAACTGTCTGCTTACATAAACAGTAATACAAGGGGTGTTATGAGCCATATTCAACGGGAAACGTCTTGCTCGAGGCCGCGATTAAATTCCAACATGGATGCTGATTTATATGGGTATAAATGGGCTCGCGATAATGTCGGGCAATCAGGTGCGACAATCTATCGATTGTATGGGAAGCCCGATGCGCCAGAGTTGTTTCTGAAACATGGCAAAGGTAGCGTTGCCAATGATGTTACAGATGAGATGGTCAGACTAAACTGGCTGACGGAATTTATGCCTCTTCCGACCATCAAGCATTTTATCCGTACTCCTGATGATGCATGGTTACTCACCACTGCGATCCCCGGGAAAACAGCATTCCAGGTATTAGAAGAATATCCTGATTCAGGTGAAAATATTGTTGATGCGCTGGCAGTGTTCCTGCGCCGGTTGCATTCGATTCCTGTTTGTAATTGTCCTTTTAACAGCGATCGCGTATTTCGTCTCGCTCAGGCGCAATCACGAATGAATAACGGTTTGGTTGATGCGAGTGATTTTGATGACGAGCGTAATGGCTGGCCTGTTGAACAAGTCTGGAAAGAAATGCATAAGCTTTTGCCATTCTCACCGGATTCAGTCGTCACTCATGGTGATTTCTCACTTGATAACCTTATTTTTGACGAGGGGAAATTAATAGGTTGTATTGATGTTGGACGAGTCGGAATCGCAGACCGATACCAGGATCTTGCCATCCTATGGAACTGCCTCGGTGAGTTTTCTCCTTCATTACAGAAACGGCTTTTTCAAAAATATGGTATTGATAATCCTGATATGAATAAATTGCAGTTTCATTTGATGCTCGATGAGTTTTTCTAATCAGAATTGGTTAATTGGTTGTAACACTGGCAGAGCATTACGCTGACTTGACGGGACGGCGGCTTTGTTGAATAAATCGAACTTTTGCTGAGTTGAAGGATCAGATCACGCATCTTCCCGACAACGCAGACCGTTCCGTGGCAAAGCAAAAGTTCAAAATCACCAACTGGTCCACCTACAACAAAGCTCTCATCAACCGTGGCTCCCTCACTTTCTGGCTGGATGATGGGGCGATTCAGGCCTGGTATGAGTCAGCAACACCTTCTTCACGAGGCAGACCTCAGCGCCCCCCCCCCCCTGCAGGTCGATCTGGTAACCCCAGCGCGGTTGCTACCAAGTAGTGACCCGCTTCGTGATGCAAAATCCGCTGACGATATTCGGGCGATCGCTGCTGAATGCCATCGAGCAGTAACGTGGCACCCCGCCCCTGCCAAGTCACCGCATCCAGACTGAACAGCACCAAGAGGCTAAAACCCAATCCCGCCGGTAGCAGCGGAGAACTACCCAGCATTGGTCCCACCAAAGCTAATGCCGTCGTGGTAAAAATCGCGATCGCCGTCAGACTCAAGCCCAGTTCGCTCATGCTTCCTCATCTAGGTCACAGTCTTCGGCGATCGCATCGATCTGATGCTGCAGCAAGCGTTTTCCATACCGGCGATCGCGCCGTCGCCCTTTCGCTGCCGTGGCCCGCTTACGAGCTCGTTTATCGACCACGATCGCATCCAAATCCGCGATCGCTTCCCAGTCCGGCAATTCAGTCTGGGGCGTCCGTTTCATTAATCCTGATCAGGCACGAAATTGCTGTGCGTAGTATCGCGCATAGCGGCCAGCCTCTGCCAACAGCGCATCGTGATTGCCTGCCTCAACAATCTGGCCGCGCTCCATCACCAAGATGCGGCTGGCATTACGAACCGTAGCCAGACGGTGAGCAATGATAAAGACCGTCCGTCCCTGCATCACCCGTTCTAGGGCCTCTTGCACCAAGGTTTCGGACTCGGAATCAAGCGCCGAAGTCGCCTCATCCAGAATTAAAATGCGTGGATCCTCTACGCCGGACGCATCGTGGCCGGCATCACCGGCGCCACAGGTGCGGTTGCTGGCGCCTATATCGCCGACATCACCGATGGGGAAGATCGGGCTCGCCACTTCGGGCTCATGAGCGCTTGTTTCGGCGTGGGTATGGTGGCAGGCCCCGTGGCCGGGGGACTGTTGGGCGCCATCTCCTTGCATGCACCATTCCTTGCGGCGGCGGTGCTCAACGGCCTCAACCTACTACTGGGCTGCTTCCTAATGCAGGAGTCGCATAAGGGAGAGCGTCGATCGACCGATGCCCTTGAGAGCCTTCAACCCAGTCAGCTCCTTCCGGTGGGCGCGGGGCATGACTATCGTCGCCGCACTTATGACTGTCTTCTTTATCATGCAACTCGTAGGACAGGTGCCGGCAGCGCTCTGGGTCATTTTCGGCGAGGACCGCTTTCGCTGGAGCGCGACGATGATCGGCCTGTCGCTTGCGGTATTCGGAATCTTGCACGCCCTCGCTCAAGCCTTCGTCACTGGTCCCGCCACCAAACGTTTCGGCGAGAAGCAGGCCATTATCGCCGGCATGGCGGCCGACGCGCTGGGCTACGTCTTGCTGGCGTTCGCGACGCGAGGCTGGATGGCCTTCCCCATTATGATTCTTCTCGCTTCCGGCGGCATCGGGATGCCCGCGTTGCAGGCCATGCTGTCCAGGCAGGTAGATGACGACCATCAGGGACAGCTTCAAGGATCGCTCGCGGCTCTTACCAGCCTAACTTCGATCACTGGACCGCTGATCGTCACGGCGATTTATGCCGCCTCGGCGAGCACATGGAACGGGTTGGCATGGATTGTAGGCGCCGCCCTATACCTTGTCTGCCTCCCCGCGTTGCGTCGCGGTGCATGGAGCCGGGCCACCTCGACCTGAATGGAAGCCGGCGGCACCTCGCTAACGGATTCACCACTCCAAGAATTGGAGCCAATCAATTCTTGCGGAGAACTGTGAATGCGCAAACCAACCCTTGGCAGAACATATCCATCGCGTCCGCCATCTCCAGCAGCCGCACGCGGCGCATCTCGGGCAGCGTTGGGTCCTGGCCACGGGTGCGCATGATCGTGCTCCTGTCGTTGAGGACCCGGCTAGGCTGGCGGGGTTGCCTTACTGGTTAGCAGAATGAATCACCGATACGCGAGCGAACGTGAAGCGACTGCTGCTGCAAAACGTCTGCGACCTGAGCAACAACATGAATGGTCTTCGGTTTCCGTGTTTCGTAAAGTCTGGAAACGCGGAAGTCAGCGCCCTGCACCATTATGTTCCGGATCTGCATCGCAGGATGCTGCTGGCTACCCTGTGGAACACCTACATCTGTATTAACGAAGCGCTGGCATTGACCCTGAGTGATTTTTCTCTGGTCCCGCCGCATCCATACCGCCAGTTGTTTACCCTCACAACGTTCCAGTAACCGGGCATGTTCATCATCAGTAACCCGTATCGTGAGCATCCTCTCTCGTTTCATCGGTATCATTACCCCCATGAACAGAAATCCCCCTTACACGGAGGCATCAGTGACCAAACAGGAAAAAACCGCCCTTAACATGGCCCGCTTTATCAGAAGCCAGACATTAACGCTTCTGGAGAAACTCAACGAGCTGGACGCGGATGAACAGGCAGACATCTGTGAATCGCTTCACGACCACGCTGATGAGCTTTACCGCAGCTGCCTCGCGCGTTTCGGTGATGACGGTGAAAACCTCTGACACATGCAGCTCCCGGAGACGGTCACAGCTTGTCTGTAAGCGGATGCCGGGAGCAGACAAGCCCGTCAGGGCGCGTCAGCGGGTGTTGGCGGGTGTCGGGGCGCAGCCATGACCCAGTCACGTAGCGATAGCGGAGTGTATACTGGCTTAACTATGCGGCATCAGAGCAGATTGTACTGAGAGTGCACCATATGCGGTGTGAAATACCGCACAGATGCGTAAGGAGAAAATACCGCATCAGGCGCTCTTCCGCTTCCTCGCTCACTGACTCGCTGCGCTCGGTCGTTCGGCTGCGGCGAGCGGTATCAGCTCACTCAAAGGCGGTAATACGGTTATCCACAGAATCAGGGGATAACGCAGGAAAGAACATGTGAGCAAAAGGCCAGCAAAAGGCCAGGAACCGTAAAAAGGCCGCGTTGCTGGCGTTTTTCCATAGGCTCCGCCCCCCTGACGAGCATCACAAAAATCGACGCTCAAGTCAGAGGTGGCGAAACCCGACAGGACTATAAAGATACCAGGCGTTTCCCCCTGGAAGCTCCCTCGTGCGCTCTCCTGTTCCGACCCTGCCGCTTACCGGATACCTGTCCGCCTTTCTCCCTTCGGGAAGCGTGGCGCTTTCTCATAGCTCACGCTGTAGGTATCTCAGTTCGGTGTAGGTCGTTCGCTCCAAGCTGGGCTGTGTGCACGAACCCCCCGTTCAGCCCGACCGCTGCGCCTTATCCGGTAACTATCGTCTTGAGTCCAACCCGGTAAGACACGACTTATCGCCACTGGCAGCAGCCACTGGTAACAGGATTAGCAGAGCGAGGTATGTAGGCGGTGCTACAGAGTTCTTGAAGTGGTGGCCTAACTACGGCTACACTAGAAGGACAGTATTTGGTATCTGCGCTCTGCTGAAGCCAGTTACCTTCGGAAAAAGAGTTGGTAGCTCTTGATCCGGCAAACAAACCACCGCTGGTAGCGGTGGTTTTTTTGTTTGCAAGCAGCAGATTACGCGCAGAAAAAAAGGATCTCAAGAAGATCCTTTGATCTTTTCTACGGGGTCTGACGCTCAGTGGAACGAAAACTCACGTTAAGGGATTTTGGTCATGAGATTATCAAAAAGGATCTTCACCTAGATCCTTTTAAATTAAAAATGAAGTTTTAAATCAATCTAAAGTATATATGAGTAAACTTGGTCTGACAGTTACCAATGCTTAATCAGTGAGGCACCTATCTCAGCGATCTGTCTATTTCGTTCATCCATAGTTGCCTGACTCCCCGTCGTGTAGATAACTACGATACGGGAGGGCTTACCATCTGGCCCCAGTGCTGCAATGATACCGCGAGACCCACGCTCACCGGCTCCAGATTTATCAGCAATAAACCAGCCAGCCGGAAGGGCCGAGCGCAGAAGTGGTCCTGCAACTTTATCCGCCTCCATCCAGTCTATTAATTGTTGCCGGGAAGCTAGAGTAAGTAGTTCGCCAGTTAATAGTTTGCGCAACGTTGTTGCCATTGCTGCAGGCATCGTGGTGTCACGCTCGTCGTTTGGTATGGCTTCATTCAGCTCCGGTTCCCAACGATCAAGGCGAGTTACATGATCCCCCATGTTGTGCAAAAAAGCGGTTAGCTCCTTCGGTCCTCCGATCGTTGTCAGAAGTAAGTTGGCCGCAGTGTTATCACTCATGGTTATGGCAGCACTGCATAATTCTCTTACTGTCATGCCATCCGTAAGATGCTTTTCTGTGACTGGTGAGTACTCAACCAAGTCATTCTGAGAATAGTGTATGCGGCGACCGAGTTGCTCTTGCCCGGCGTCAACACGGGATAATACCGCGCCACATAGCAGAACTTTAAAAGTGCTCATCATTGGAAAACGTTCTTCGGGGCGAAAACTCTCAAGGATCTTACCGCTGTTGAGATCCAGTTCGATGTAACCCACTCGTGCACCCAACTGATCTTCAGCATCTTTTACTTTCACCAGCGTTTCTGGGTGAGCAAAAACAGGAAGGCAAAATGCCGCAAAAAAGGGAATAAGGGCGACACGGAAATGTTGAATACTCATACTCTTCCTTTTTCAATATTATTGAAGCATTTATCAGGGTTATTGTCTCATGAGCGGATACATATTTGAATGTATTTAGAAAAATAAACAAATAGGGGTTCCGCGCACATTTCCCCGAAAAGTGCCACCTGACGTCTAAGAAACCATTATTATCATGACATTAACCTATAAAAATAGGCGTATCACGAGGCCCTTTCGTCTTCAAGAATT
